# Supplementary material for: Needs of informal caregivers of people with a rare disease: a rapid review of the literature
Source: BMJ Open. 2022 Dec 12;12(12):e063263. doi: 10.1136/bmjopen-2022-063263 (PMC9748923; doi:10.1136/bmjopen-2022-063263)
Supplement: Supplementary data [file bmjopen-2022-063263supp001.pdf]

**, Supplementary file 1: Detailed search strategy**

| |
|---|
| ‘informal caregiver*’ AND ‘rare disease*’ |

Non- English articles and duplicates were excluded
