# Supplementary material for: Needs of informal caregivers of people with a rare disease: a rapid review of the literature
Source: BMJ Open. 2022 Dec 12;12(12):e063263. doi: 10.1136/bmjopen-2022-063263 (PMC9748923; doi:10.1136/bmjopen-2022-063263)
Supplement: Supplementary data [file bmjopen-2022-063263supp002.pdf]

Supplementary file 2: Data extraction table

Author & year of publication	Country in which the study was conducted	Data collection method(s)	Participants	Identified caregiver needs
Anderson, M., Elliott EJ, Zurynski YA.<br><br>2009	Australia	Survey	Parents/carers	• Support around diagnosis.<br>• Access to peer support groups.<br>• Psychological support.
Applebaum A.J., Polacek L.C., Walsh L. *et al.*<br><br>2020	USA	Survey and semi-structured interview	Caregivers of patients with Erdheim-Chester disease	• Social connections to prevent extreme isolation
Aubeeluck A.V. Buchanan H.E., Stupple E.J.N.<br><br>2011	England	Focus groups	Caregivers of those with Huntington's Disease	• More time to focus on themselves.
Craig T.J., Banjerji A., Riedl M.A., *et al.*<br><br>2021	USA	Online survey	Adults caring for an individual with Hereditary angioedema (HAE)	• Recognition for the important role they have.<br>• Inclusion in treatment discussions.
Hanbury A, Smith AB & Buesch K<br><br>2021	England	Vignettes were developed	Caregivers	• Engagement with research
Baumbusch, J., Mayer, S., & Sloan-Yip, I.<br><br>2018	Canada	Semi-structured interviews	Parents of children with a RD	• Acknowledgment from healthcare providers<br>• Support coordinating care<br>• Peer support – information and emotional<br>• Policies and programs to validate their role<br>• Support from genetic counsellor to connect them to others

Bendixen, R. M., & Houtrow, A.<br><br>2017	USA	Interviews	Parents of children with Duchenne muscular dystrophy	• Listened to by healthcare providers<br>• Inclusion in the diagnostic process<br>• Improved delivery of the diagnosis<br>• Improved follow up after diagnosis
Cañedo-Ayala, M., Rice, D. B., Levis, B., et al.<br><br>2020	USA	Questionnaire	Informal caregivers	• Mental health support
Currie, G., & Szabo, J.<br><br>2019	Canada	Semi-structured interviews	Parents of children with RDs	• Communication issues need addressed as parents often know more about the disease than healthcare providers.<br>• Improved coordination of care between providers and services caring for children with rare diseases.<br>• Gap in accessibility to government supports needs addressed.
Currie, G., & Szabo, J.<br><br>2019	Canada	Semi-structured interviews	Parents of children with rare neurodevelopmental diseases	• Health-care providers need more understanding of rare conditions.<br>• More support needed, parents often 'lose themselves' in this demanding caring role.
Flores D., Ribate M.P., Montolio M., *et al.*<br><br>2020	Spain	Questionnaires	Caregivers to patients with Duchenne muscular dystrophy	• Financial support.
Kanters T.A., van der Ploeg Ans. T, Brouwer W.B.F., *et al.*<br><br>2013	Netherlands	Questionnaire	Caregivers for those with Pompe disease.	• Support needed for informal caregivers.
Kasparian, N. A., Rutstein, A., Sansom-Daly, *et al.*	Australia	Telephone interviews	Patients and caregivers affected by Von Hippel-Lindau disease	• More supportive care services requested.

2015				
Landfelt E., Lindgren P., Bell C.F., *et al.*<br><br>2016	Germany, Italy, UK, USA	Visual analogue scale, survey, interview	Caregivers to patients with Duchenne muscular dystrophy	• Screening for depression.<br>• Holistic approach to family mental health needed.
Lagae, L., Irwin, J., Gibson, E., *et al.*<br><br>2019	Europe	Survey	Caregivers for those with Dravet syndrome (DS)	• Time needed to escape caring duties.<br>• Formal support and respite required.<br>• Stress is common – support needed.
Lopez-Bastida J., Pena-Longobardo L.M., Aranda-Reneo I, *et al.*<br><br>2017	Spain	Questionnaire	Patients and caregivers with Spinal Muscular Atrophy	• Financial support<br>• QoL must be addressed
Lyon, M. E., Thompkins, J. D., Fratantoni, K., *et al.*<br><br>2019	USA	Semi-structured interviews	Caregiving families	• Worries about the future need to be addressed.<br>• More time for themselves needed.<br>• Financial concerns must be addressed.
McKnight, A. J. M., Walker, R., Collins, C. (2020).	Northern Ireland	Report	N/A	• Access to accurate information<br>• Access to appropriate services<br>• Improved communication
McMullan, J., Crowe, A. L., Bailie, C. *et al.*<br><br>2020	Northern Ireland	Survey and semi-structured interviews	Rare disease collaborative groups	• Caregivers often overlooked in RD research – their opinions and experiences must be valued.
McMullan J, Crowe A.L., Downes K., *et al.*<br><br>2021	Northern Ireland	Survey and workshop	Caregivers of those with a rare disease	• Improved interactions with healthcare professionals.<br>• Improved emotional, psychological and social support.<br>• Assistance with finances.<br>• Better awareness of support services.
Mooney J., Graham K. & Watts R.A.	England	Semi-structured interviews	Patients with ANCA-associated vasculitis and their caregivers.	• Emotional support is needed.<br>• Reassurance about the future.

2019				
Mori, Y., Downs, J., Wong, K., *et al.*<br><br>2017	Australia	Survey	Families with a child with CDKL5 disorder	• Burden of daily caregiving requires support, particularly in relation to emotional wellbeing, sleep problems, financial difficulties, QoL.
Mutch, K., Methley, A., Hamid, S., *et al.*<br><br>2017	England	Semi-structured interviews	Partners of people with NMO	• Acknowledgement from HCP regarding the vital role they play in caring.
Palacios-Cena, D., Famoso-Perez, P., Salom-Moreno, J., *et al.*<br><br>2018	Spain	Interviews, focus groups, researcher's field notes, caregiver's personal documents	Caregivers of children with Rett Syndrome	• Answers needed regarding 'the first symptoms' and 'the need for a diagnosis'.<br>• Help with managing day to day life.<br>• Financial support.
Pelentsov, L. J., Fielder, A. L., & Esterman, A. J.<br><br>2016	Australia	Semi-structured focus group interviews	Parents of a child with a RD	• Social isolation must be addressed.<br>• Knowledge of HCP needs to be improved.<br>• Support needed as family relationship often impacted due to demands of caring.
Pelentsov, L. J., Laws, T. A., & Esterman, A. J.<br><br>2015	Australia	Scoping Review	Parents of child with a RD	• Improved parental supportive care – common unmet needs between RDs.
Rice D.B., Canedo-Ayala M., Carboni-Jimeniez A., Carrier M-E, *et al.*<br><br>2020	USA	Online questionnaire	Caregivers of people with SSc	• Emotional support is required.<br>• Help with physical needs.<br>• Interventions needed delivered through hardcopy or online resources, including those delivered after the care recipient's diagnosis.
Rice D.B., Carbino-Jimeniz A., Canedo-Ayala M. *et al.*<br><br>2020	USA	Scoping review	N/A	• Psychosocial interventions needed to reduce caregivers stress, burden and feelings of isolation among caregivers.

				• Future research should design interventions for caregivers.
Rodriguez A.A., Martinez O., Amayra I, *et al.*<br><br>2021	Spain	Questionnaire	Carers of children with neuromuscular disease	• Financial assistance.<br>• Employment support.<br>• Physical and psychological support.
Selman L.E., Beynon T., Radcliffe S., *et al.*<br><br>2014	London	Semi-structured qualitative interviews	Adult informal caregivers of patients with Cutaneous T-cell lymphoma	• Easily accessible services are needed that include the family in the unit of care, provide support and information, and understand the process of family adjustment and adaptation.
Sloper, T., & Beresford, B.<br><br>2006	UK	Report	Families of disabled children	• Policies and structures to support social and economic needs.
Somanadhan, S., & Larkin, P. J.<br><br>2016	Ireland	In-depth interviews	Parents of those with Mucopolysaccharidosis	• Reassurance and certainty needed about the future.
Wiblin, L., Durcan, R., Lee, M., *et al.*<br><br>2017	England	Qualitative in depth interviews	Patients and caregivers living with MSA and PSP	• Better connections to others to reduce social isolation.<br>• Improved communication.
Williams, J. K., Skirton, H., Paulsen, J. S., *et al.*<br><br>2009	USA, Canada	Focus groups	Adult caregivers of people with Huntington's disease	• Emotional distress support should be provided.<br>• Assistance in managing several roles.<br>• Mental health monitoring.
Wu, Y., Al-Janabi, H., Mallett, A., *et al.*<br><br>2020	Australia	Clinical data was used from Mitochondrial Disease, Epileptic	Parents of those with rare genetic conditions	• Health effects on family members need to be considered.

		Encephalopathy and Brain Malformation projects		
