# Supplementary material for: Needs of informal caregivers of people with a rare disease: a rapid review of the literature
Source: BMJ Open. 2022 Dec 12;12(12):e063263. doi: 10.1136/bmjopen-2022-063263 (PMC9748923; doi:10.1136/bmjopen-2022-063263)
Supplement: Author's manuscript [file bmjopen-2022-063263.draft_revisions.pdf]

BMJ Open is committed to open peer review. As part of this commitment we make the peer review history of every article we publish publicly available.

When an article is published we post the peer reviewers' comments and the authors' responses online. We also post the versions of the paper that were used during peer review. These are the versions that the peer review comments apply to.

The versions of the paper that follow are the versions that were submitted during the peer review process. They are not the versions of record or the final published versions. They should not be cited or distributed as the published version of this manuscript.

BMJ Open is an open access journal and the full, final, typeset and author-corrected version of record of the manuscript is available on our site with no access controls, subscription charges or pay-per-view fees (http://bmjopen.bmj.com).

If you have any questions on BMJ Open's open peer review process please email info.bmjopen@bmj.com

BMJ Open

The needs of informal caregivers of people with a rare disease: a rapid review of the literature.

Journal:	*BMJ Open*
Manuscript ID	bmjopen-2022-063263
Article Type:	Original research
Date Submitted by the Author:	02-Apr-2022
Complete List of Authors:	Mcmullan, Julie; Queen's University Belfast Faculty of Medicine Health and Life Sciences, Centre for Public Health<br>Lohfeld, Lynne; Wenzhou Medical University Eye Hospital, ; Queen's University Belfast, Centre for Public Health<br>McKnight, Amy; Queen's University Belfast, Centre for Public Health
Keywords:	Quality in health care < HEALTH SERVICES ADMINISTRATION & MANAGEMENT, Health policy < HEALTH SERVICES ADMINISTRATION & MANAGEMENT, PUBLIC HEALTH

SCHOLARONE™
Manuscripts

I, the Submitting Author has the right to grant and does grant on behalf of all authors of the Work (as defined in the below author licence), an exclusive licence and/or a non-exclusive licence for contributions from authors who are: i) UK Crown employees; ii) where BMJ has agreed a CC-BY licence shall apply, and/or iii) in accordance with the terms applicable for US Federal Government officers or employees acting as part of their official duties; on a worldwide, perpetual, irrevocable, royalty-free basis to BMJ Publishing Group Ltd ("BMJ") its licensees and where the relevant Journal is co-owned by BMJ to the co-owners of the Journal, to publish the Work in this journal and any other BMJ products and to exploit all rights, as set out in our licence.

The Submitting Author accepts and understands that any supply made under these terms is made by BMJ to the Submitting Author unless you are acting as an employee on behalf of your employer or a postgraduate student of an affiliated institution which is paying any applicable article publishing charge ("APC") for Open Access articles. Where the Submitting Author wishes to make the Work available on an Open Access basis (and intends to pay the relevant APC), the terms of reuse of such Open Access shall be governed by a Creative Commons licence – details of these licences and which Creative Commons licence will apply to this Work are set out in our licence referred to above.

Other than as permitted in any relevant BMJ Author's Self Archiving Policies, I confirm this Work has not been accepted for publication elsewhere, is not being considered for publication elsewhere and does not duplicate material already published. I confirm all authors consent to publication of this Work and authorise the granting of this licence.

For peer review only

The needs of informal caregivers of people with a rare disease: a rapid review of the literature.

Authors

Julie McMullan (PDRF) [1], Lynne Lohfeld (Lecturer) [1], Amy Jayne McKnight (Professor) [1]

Institutional address

[1] Centre for Public Health, School of Medicine Dentistry and Biomedical Sciences, Institute of Clinical Science Block A, Grosvenor Road, Belfast, BT12 6BA.

Corresponding author

Julie McMullan

Email: raredisease@qub.ac.uk

Abstract

Objectives: Many people living with a rare disease are cared for by a family member. Due to a frequent lack of individual rare disease (RD) knowledge from healthcare professionals, the patient and their informal caregiver are frequently obliged to become 'experts' in their specific condition. This puts huge strain on family life and results in caregivers juggling multiple roles in addition to unique caring roles including as advocate, case manager, and medical navigator. We conducted a rapid review of literature reporting on the unmet needs of informal caregivers for people living with a RD.

Setting: Searches were conducted in Medline, Embase, Web of Science, GreyLit and OpenGrey.

Results: Thirty-five papers were included in the final review & data extracted. This rapid review presents several unmet needs identified by informal caregivers of persons with a RD. The related literature was organised thematically: caregiver burden, support through the diagnosis process, social needs, financial needs, psychological needs, information needs and acknowledgement from healthcare professionals.

Conclusions: This review provides evidence that increased meaningful support is required for caregivers. Active engagement should be encouraged from this cohort in future research and awareness raised of the support available to improving the quality of life for families living with an RD.

For peer review only

The unmet needs identified through this review will benefit people living with a RD, caregivers, healthcare professionals and policy makers.

Key words: burden, informal caregiver, needs, rare disease, review.

Introduction

Rare diseases (RDs) are those which affect less than 1 in 2000 people in a specified population [1]. Although each RD occurs infrequently, collectively RDs are a major public health issue affecting approximately 300 million people globally [2 3]. RDs result in a wide variety of healthcare needs stemming from the involvement of multi-organ systems and cognitive and/or developmental issues. Many RDs are chronic, complex and associated with physical, intellectual, or neurological disabilities that significantly affect patients and their families. In addition, many families living with a RD lack peer and community support services [4-7].

The family often plays a pivotal role in a person's adjustment to chronic disease and is influenced by behavioural, social factors [8]. Caring for someone with an RD affects many areas of life including psychologically, economically, physically and logistically [9]. The importance of good mental and physical health for the informal caregiver is therefore vital for their own wellbeing and to ensure they can sustain the essential role of assisting the person with an RD [10 11 11]. Individuals with RDs and their families often have limited evidence-based information to guide decisions about disease management and symptom relief [12 13]. Further, the inherent uncertainty that comes with having a RD, including delays in diagnosis and a lack of knowledge about current and future care needs [14 15], impact access to services and management of the RD [16]. Research on the experience of having an RD indicates that care and service needs are often not identified based on the severity of the health condition, but rather are associated with poor quality of care and barriers to access [15 17].

Given the few people with a specific RD, healthcare providers often are not knowledgeable about the condition. Therefore people living with a RD and their carers have to become their own experts [18]. This causes a change to the usual patient-doctor relationship, which can bring challenges [19 20]. Caring for someone with a RD can be highly demanding often requiring intense and unique care specific to the individuals needs [21]. Delayed diagnosis, lifelong caring, limited capacity for independent living, lack of treatment options and large health service needs have severe impacts on parent's physical and psychosocial wellbeing [22].

Rapid reviews are an emerging type of knowledge synthesis used to inform health-related policy decisions and discussions, especially when information needs are immediate [23-26]. Rapid reviews streamline systematic review methods – for example, by focusing the literature search [23] while still aiming to produce valid conclusions. The requirements or the review, which was undertaken with a short deadline, were for a short but in-depth synthesis of the current state of the issues facing caregivers for those with a rare disease.

The purpose of this study was to identify literature reporting on the unmet needs of informal caregivers for people living with a RD.

Methodology

Search strategy and inclusion criteria

Three electronic databases were searched -- Medline, Embase and Web of Science -- using the combined terms 'informal caregiver*' and 'rare disease*'. All searches were conducted on 14 September 2021. Reference lists of included papers were screened for further sources. A search was also conducted of grey literature using the databases GreyLit and OpenGrey. Duplicates and non-English language articles were excluded.

Study selection and data extraction

Database searches were last conducted on 14 September 2021 by JM. Titles and abstracts of the identified articles were downloaded onto Endnote. Duplicate articles were removed and the remaining papers were screened through analysing their titles and abstracts. If relevant, the papers were then further screened by reading the full text. Data were extracted by JM who recorded the follow data for each study:

- Author & year of publication
- Country in which the study was conducted
- Data collection method(s)
- Participants
- Identified caregiver needs

For peer review only

The criteria for inclusion were articles that address caregiving for people living with an RD. Articles on caregiving alone or RD alone were excluded. Primary research studies and systematic review were considered for inclusion as shown in Table 1.

Table 1: Literature search results

Database	No. of articles retrieved	No. of articles included in review
Medline	6	5
Embase	6	5
Web of Science	62	13
GreyLit	0	0
OpenGrey	0	0

This table shows the number of articles identified from each database before reference lists were checked.

An illustration of the search strategy including databases searched and screening methods is displayed in Figure 1, modelled on the PRISMA flow diagram.

Patient and Public Involvement

The need for this review was highlighted by a priorities workshop in 2020, which considered contributions from >2,000 individuals living with rare disease. The review was also shared with representatives from a local rare disease charity in Northern Ireland (the Northern Ireland Rare Disease Partnership).

Results

General description of the literature

Sources initially identified from each database were as follows: MEDLINE *n= 6*, Embase *n= 6*, Web of Science *n= 62*, OpenGrey *n=0* and GreyLit *n=0*. The screening process resulted in 30 documents which were reviewed based on their titles and abstracts. Following the screening 5 duplicate papers were removed and 25 papers were identified for full text screening. Further studies were identified from search the reference lists. Finally, 35 texts were included in the full review, with characteristics of each source summarised from the completed data extraction table (Supplementary file 1).

Of the texts included, publication dates were from 2006 to 2021 and the studies were conducted in Australia, Canada, Germany, Ireland, Italy, Netherlands, Spain, United Kingdom and the United States.

A narrative review is presented below, highlighting the main needs of caregivers of people living with an RD. The related literature has been organised thematically under the following headings:

- Caregiver burden
- Support through the diagnosis process
- Social needs
- Financial needs
- Psychological needs
- Information needs
- Acknowledgement from healthcare professionals

Findings from these studies were organised thematically under the following headings as shown visually in Figure 2.

Caregiver burden

Caring for someone with an RD presents a range of challenges [27 28] that are affected by the severity of the illness and its duration, knowledge about the condition and its changes over time, and one's ability to address the emotional toll involved with long-term care [29]. Despite the stress and difficulties associated with being a family caregiver [30], often they acknowledge the positive aspects of caring for their loved ones [30].

The quality of life of caregivers is often compromised by their placing others' needs before their own [31]. Previous research has shown that family caregivers do not have enough time for themselves, [32] and that caring for someone with an RD can negatively impact on all dimensions of family life [33]. Caregivers are often forced to miss work or school days due to the demands of their role, frequently address unpleasant events and watch a loved one suffer [28]. Consequently, they would benefit greatly from support to reduce the burden of caring [34]. This includes both supports for informal carers of people with a variety of health issues, as well as for RD-related issues [34].

Support through the diagnosis process

In spite of marked medical progress over the past several decades in identifying an RD, there are substantial delays in diagnosing these conditions [35]. This can have adverse impacts on families [12]. Little attention is given to parent concern in the diagnostic process with previous research reporting

For peer review only - http://bmjopen.bmj.com/site/about/guidelines.xhtml

concerns regarding how the diagnosis was delivered to parents, lack of guidance and poor follow-up post diagnosis [35]. Previous research has shown that families are often dissatisfied by the way in which a diagnosis is given, including an insensitive style of communication, not offering support or counselling, and inadequate provision of information about the disease. This can cause emotional stress for caregivers who have not yet learned about the condition and how it will affect them and their families. Therefore, interventions to support the following a diagnosis would be useful, delivered either in hardcopy or online [36].

Social needs

Social isolation is common among caregivers with the impact on personal relationships being a reoccurring theme [4 5]. All too often, caregivers have little time to themselves and rely heavily on family members for support and respite. This in turn causes further emotional stress and in the case of parents, uncertainty about their child's future [37]. Parents of children with an RD often feel isolated from mainstream society and struggle to stay socially connected [4]. It was suggested that the insights gained through research studies regarding the impact on caregivers' social lives should be considered in future clinical service planning. Additionally holistic, empathic, and person-centred medical and psychosocial care is urgently needed for this cohort [6]. Support is needed as family relationships are often impacted due to demands of caring [4]. There is a need for improved parental supportive care as many common unmet needs exist across RDs [38].

Financial needs

Previous research has indicated that financial issues are a top concern of caregivers of persons with RD [30 6 39 27]. The high economic costs that families must cover means that financial burden is common among caregivers of those with an RD, many of whom are forced to exhaust their financial savings [40]. Purchasing equipment, hiring professionals, and the additional financial burden the illness has on families poses immense stress on family life [41]. Often financial issues impact on the ability of the caregiver to meet the individual's healthcare needs, increasing the strain on their lives [42]. Substantial social/economic burden is mostly attributable to high direct non-healthcare costs [43]. Previous research has shown that caregivers' financial burden might be conditioned by the clinical condition of the patient [44]. In addition to the direct healthcare costs, caregivers of those with an RD report that their career choices are influenced due to their caring role. Furthermore, caring duties can result in missed working days due to emergency caring demands [37] making it difficult for many caregivers to maintain their job and advance in their careers [41]. Financial hardship adversely effects the mental health of caregivers [42].

For peer review only

Psychological needs

There is a lack of psychological support for families caring for children with a RD, and many report that accessing appropriate psychological care is difficult [6 12]. Previous research has suggested that this form of support should be offered at the time of diagnosis [12 10]. Depressive symptoms are often associated with caregiving burden and therefore there is a need to develop interventions for caregivers considering their special needs [10]. Caregivers often express concerns about the future [32], experiencing emotional distress that can compromise the well-being of family carers, who attempt to maintain multiple roles [11]. Psychological interventions can help reduce stress, a sense of being burdened and feelings of isolation that many RD caregivers feel [45]. Research has suggested that screening for depression is needed and emphasises the need for a holistic approach to family mental health in the context of chronic childhood disease [46]. Peer support is a key resource in terms of information and emotional support for parents who often begin their journey feeling isolated and alone [21].

Information needs

Suggestions were made that information about the condition should be given to caregivers at the time of diagnosis as well as signposting them to groups connecting families who share common experiences [21]. Caregivers require access to accurate information, appropriate services and improved communication between patients, families, and a range of both health and social care and other public services [2]. Families require easily accessible services that include the family in the unit of care, provide support and information, and understand the process of family adjustment and adaptation in the long term [47]. Policies and structures are needed to support social and economic needs [48]. Engaging caregivers in future avenues of research is vital to ensure resources and funding are targeted in the best way [49].

Acknowledgement from healthcare professionals

Out of necessity, informal carers often know more about a particular RD than healthcare providers do. This often leads to poor communication and collaboration. Furthermore a lack of coordination of care force carers to fill the gap by juggling multiple roles including that of advocate, case manager, and medical navigator [50]. Caregivers often experience silencing or being silenced when interacting with health-care and social care systems and providers [51]. This has been attributed to the lack of knowledge about RDs by health-care providers who also are unaware of the impact that caring for someone with RD [50]. This points to the need to improve the knowledge of health care providers on the medical, social and financial impact these informal carers experience [4]. Health care providers

For peer review only

should also acknowledge the vital role that informal caregivers play in promoting the health and quality of life of persons with an RD [52]. Caregivers, especially parents caring for someone with an RD, work hard to be heard and acquire services within health and social systems [50]. Another way that informal caregivers are silenced is due to how they are overlooked in the world of research despite growing emphasis on ensuring 'patient and public involvement' (PPI). It is vital that this pattern is changed so that they have an opportunity to be heard [53 27].

Discussion

The purpose of this rapid review was to synthesise and describe what is currently known about the needs of informal caregivers for people living with an RD. Based on the findings, several recommendations for future healthcare practices and policies, as well as for research are evident. First, it is important to consider the extreme burden often experienced by these caregivers, many of whom place the need of others ahead of their own while navigating this journey with little or no support s [31]. Support is needed to help caregivers with many aspects of their caring duties from the initial diagnosis process which has been a difficult time for many families [35].

Social support would be welcomed to enable caregivers to have much needed time away from their caring duties to recharge and also to relieve them of the emotional strain which impacts on not only themselves but their wider family [4]. The mental strain of caregiving is evident from previous research, psychological interventions are required that consider the family as a whole to reduce the emotional strain and depression too often experienced by caregivers [21 45]. Many caregivers have also reported struggling financially not only due to the high costs of the practical side of caring but as a result of the impact caring can have on their ability to maintain a career [41], financial support therefore a priority.

Clear information should be provided for caregivers from diagnosis through to their rare disease journey [2] to replace the current situation of confusion and uncertainty due to unclear and incomplete or conflicting messages. Lastly, caregivers must be recognised by healthcare professionals for the integral part they have in caring for someone with a rare disease. This relationship often changes the dynamic between health professional and patient and so caregivers must be listened to and viewed as an 'expert' [52]. All of the above-mentioned issues should be taken into consideration when planning future research, it is vital that informal caregivers views are valued.

Strengths and limitations

For peer review only

This study explored a cohort of individuals who frequently go unnoticed and unreported in the literature – informal caregivers of individuals diagnosed with a rare disease. The rapid review followed the guidelines for conducting a rapid review of the literature [23 24 25 26]. The review was conducted by experienced researchers in the area of RD carer needs who have a wealth of relevant experience. Additionally, manual searches of reference lists were conducted to identify any papers not found by the initial search strategy.

However, it is important to acknowledge limitations of this review. Due to resource constraints, only one author (JM) initially screened the titles and abstracts from the total set of documents retrieved. This could lead to bias. However, conducting a rapid review produced results quickly, including identifying the main self-identified needs of caregivers of people living with an RD. This is particularly important given the key role that informal caregivers play, and the fact that earlier research has focused on the person with an RD and not the caregiver. The authors of this paper consider the needs of RD caregivers a priority and recognises the value they bring to the unique caring needs.

Conclusion

This rapid review presents several unmet needs identified by informal caregivers of persons with a RD. It is hoped that the findings contribute to increasing the amount of meaningful support for caregivers as well as encourage their active participation in improving the quality of life for families living with an RD. It is important that this cohort are engaged in future research to ensure their needs are being addressed. Better meeting needs of informal caregivers for people living with a RD is in the best interests of people with a RD, healthcare professionals and policy makers, as well as caregivers. It is important that awareness is raised about the range of support options are available for informal caregivers of people with an RD from health and social care providers, charities and/or support groups [27].

Abbreviations

RD Rare disease

Declarations

- Ethics approval and consent to participate: Not applicable
- Consent for publication: Not applicable
- Availability of data and materials: Not applicable
- Competing interests: The authors declare that they have no competing interests.

For peer review only

- Funding: JM was supported by an award from the NI Public Health Agency and the Medical Research Council (MC_PC_16018) – Northern Ireland Executive support of the Northern Ireland Genomic Medicine Centre though Belfast Health and Social Care Trust.
- Authors' contributions: JM contributed to data curation, formal analysis, data interpretation, drafting and reviewing the original manuscript. LL & AJM contributed to study conception and design, data interpretation, project administration, supervision and reviewing manuscript drafts. All authors approved the final version of the manuscript for submission.
- Acknowledgements: Not applicable

For peer review only

Reference list

1. Beaulieu CL, Majewski J, Schwartzentruber J, et al. FORGE Canada Consortium: outcomes of a 2-year national rare-disease gene discovery project. *American Journal of Human Genetics* 2014;94(6):809-17.
2. McKnight A, Walker R, Collins C. Communications and Information Review: NI Report for Rare Diseases, 2020.
3. McMullan J, Crowe A, Kerr K, et al. Perspectives on a NI Rare Disease Registry, 2020.
4. Pelentsov LJ, Fielder AL, Esterman AJ. The supportive care needs of parents with a child with a rare disease: A qualitative descriptive study. *Journal of Pediatric Nursing* 2016;31(3):e207-e18.
5. Wiblin L, Durcan R, Lee M, et al. The importance of connection to others in QoL in MSA and PSP. *Parkinsons Disease* 2017:1-9.
6. Kasparian NA, Rutstein A, Sansom-Daly UM, et al. Through the looking glass: An exploratory study of the lived experiences and unmet needs of families affected by Von Hippel-Lindau disease. *European Journal of Human Genetics* 2015;23(1):34-40.
7. Kole A, Faurisson F. The Voice of 12,000 Patients: Experiences and expectations of rare disease patients on diagnosis and care in Europe. Eurordis2009 [Available from: http://www.eurordis.org/publication/voice-12000-patients accessed 9 September 2021.
8. Buchanan H, Huang C. Health-related quality of life among informal caregivers assisting people with multiple sclerosis. *Disability and Rehabilitation* 2011;33(2):113-21.
9. Mulroy S, Robertson L, Aiberti K, et al. The impact of having a sibling with an intellectual disability: Parental perspectives in two disorders. *J Intellect Disabil Res* 2008;52:216-29.
10. Canedo-Ayala M, Rice DB, Levis B, et al. Factors associated with symptoms of depression among informal caregivers of people with systemic sclerosis: A cross-sectional study. *Disability and Rehabilitation* 2020;42(3):394-99.
11. Williams JK, Skirton H, Paulsen JS, et al. The emotional experiences of family carers in Huntington disease. *Journal of Advanced Nursing* 2009;65(4):789-98.
12. Anderson M, Elliott EJ, Zurynski YA. Australian families living with rare disease: experiences of diagnosis, health services use and needs for psychosocial support. *Orphanet J Rare Dis* 2013;8:22. doi: 10.1186/1750-1172-8-22 [published Online First: 2013/02/13]
13. Forsythe LP, Szydiowski V, Murad MH, et al. A systematic review of approaches for engaging patients for research on rare diseases. *Journal of General Internal Medicine* 2014;29(53):788-800.
14. Grut L, Kvam MH. Facing ignorance: people with rare disorders and their experiences with public health and welfare services. *Scandinavian Journal of Disability Research* 2013;15(1):20-32.

15. Huyard C. What if anything is specific about having a rare disorder? Patients' judgements on being ill and being rare. *Health Expectations* 2009;12(4)
16. Delive L, Samuelsson L, Tallborn A, et al. Stress and well-being among parents of children with rare diseases: a prospective intervention study. *Journal of Advanced Nursing* 2006;53(4):392-402.
17. Farmer JE, Marien WE, Clark MJ, et al. Primary care supports for children with chronic health conditions: identifying and predicting unmet family needs. *Journal of Pediatric Psychology* 2004;29(5):355-67.
18. Crowe A, McKnight A, McAneney H. Communication needs for individuals with rare diseases within and around the healthcare system of Northern Ireland. *Frontiers in Public Health* 2019;7(236)
19. Budych K, Helms TM, C. S. How do patients with rare diseases experience the medical encounter? Exploring the role behavior and its impact on patient-physician interaction. *Health Policy* 2012;105(2-3):154-64.
20. Dudding-Byth T. A powerful team: The family physican advocating for patients with a rare diease. *Australian Family Physician* 2015;44(9):634-38.
21. Baumbusch J, Mayer S, Sloan-Yip I. Alone in a crowd? Parents of children with rare diseases' experiences of navigating the healthcare system. *Journal of Genetic Counseling* 2018;28(1):80-90.
22. Madeo AC, K.E. OB, Bernhardt BA, et al. Factors associated with perceived uncertainty among parents of children with undiagnosed medical conditions. *American Journal of Medical Genetics* 2012;158A(8):1887-84.
23. Watt A, Cameron A, Sturm L, et al. Rapid reviews verus full systematic reviews: an inventory of current methods and practice in health technology assessment. . *International Journal of Technology Assessment in Health Care* 2008;24:133-39.
24. Ganann B, Ciliska D, Thomas H. Expediting systematic reviews: methods and implications of rapid reviews. . *Implementation Science* 2010;5(56)
25. Khangura S, Konnyu K, Cushman R, et al. Evidence summaries: the evolution of a rapid review approach. *Systematic reviews* 2012;1(10)
26. Konnya K, Kwok E, Skidmore B, et al. The effectiveness and safety of emergency department short stay units: a rapid review. *Open Medicine* 2012;6(e10-e16)
27. McMullan J, Crowe A, Downes K, et al. Carer reported experiences: Supporting someone with a rare disease. *Health and Social Care in the Community* 2021;0:1-12.
28. Craig TJ, Banerji A, Riedl MA, et al. Caregivers' role in managing hereditary angioedema and perceptions of treatment-related burden. *Allergy and Asthma Proceedings* 2021;42:S11-S16. doi: 10.2500/aap.2021.42.210029
29. Mooney J, Graham K, Watts RA. Impact of caring for someone with a rare rheumatic condition, views from patients and informal carers-the need for cat-like vigilance. *Rheumatology Advances in Practice* 2019;3(1) doi: 10.1093/rap/rkz003
30. Kanters TA, van der Ploeg AT, Brouwer WBF, et al. The impact of informal care for patients with Pompe disease: An application of the CarerQol instrument. *Molecular Genetics and Metabolism* 2013;110(3):281-86. doi: 10.1016/j.ymgme.2013.07.020
31. Aubeeluck AV, Buchanan H, Stupple EJN. 'All the burden on all the carers': Exploring quality of life with family caregivers of Huntington's disease patients. *Quality of Life Research* 2012;21(8):1425-35.
32. Lyon ME, Thompkins JD, Fratantoni K, et al. Family caregivers of children and adolescents with rare diseases: A novel palliative care intervention. *BMJ Supportive and Palliative Care* 2019;25(25)
33. Somanadhan S, Larkin PJ. Parents' experiences of living with, and caring for children, adolescents and young adults with Mucopolysaccharidosis (MPS). . *Orphanet J Rare Dis* 2016;11(1):138.

For peer review only

34. Wu Y, Sun F, Lee P. Family caregivers' lived experiences of caring for epidermolysis bullosa patients: A phenomenological study. *Journal of Clincial Nursing* 2020;29(9-10):1552-60.
35. Bendixen RM, Houtrow A. Parental reflections on the diagnostic process for duchenne muscular dystrophy: A qualitative study. *Journal of Pediatric Health Care* 2017;31(3):285-92.
36. Rice DB, Canedo-Ayala M, Carboni-Jimenez A, et al. Challenges and support service preferences of informal caregivers of people with systemic sclerosis: a cross-sectional survey. *Disability & Rehabiliation* 2020;42(16):2304-10.
37. Lagae L, Irwin J, Gibson E, et al. Caregiver impact and health service use in high and low severity Dravet syndrome: A multinational cohort study. *Seizure* 2019;65:72-77.
38. Pelentsov LJ, Laws TA, Esterman AJ. The supportive care needs of parents caring for a child with a rare disease: A scoping review. *Disability and Health Journal* 2015;8(4):475-91.
39. Rodriguez AA, Martinez O, Amayra I, et al. Diseases Costs and Impact of the Caring Role on Informal Carers of Children with Neuromuscular Disease. *International Journal of Environmental Research and Public Health* 2021;18(6) doi: 10.3390/ijerph18062991
40. Applebaum AJ, Polacek LC, Walsh L, et al. The unique burden of rare cancer caregiving: caregivers of patients with Erdheim-Chester disease. *Leukemia Lymphoma* 2020;61(6):1406-17.
41. Palacios-Cena D, Famoso-Perez P, Salom-Moreno J, et al. "Living an Obstacle Course": A qualitative study examining the experiences of caregivers of children with Rett Syndrome. *Int J Environ Res Public Health* 2018;16(1):25.
42. Mori Y, Downs J, Wong K, et al. Impacts of caring for a child with the CDKL5 disorder on parental wellbeing and family quality of life. *Orphanet J Rare Dis* 2017;12(1):16.
43. Lopez-Bastida J, Pena-Longobardo LM, Aranda-Reneo I, et al. Social/economic costs and health-related quality of life in patients with spinal muscular atrophy (SMA) in Spain. *Orphanet Journal of Rare Diseases* 2017;12 doi: 10.1186/s13023-017-0695-0
44. Flores D, Ribate MP, Montolio M, et al. Quantifying the economic impact of caregiving for Duchenne muscular dystrophy (DMD) in Spain. *European Journal of Health Economics* 2020;21(7):1015-23. doi: 10.1007/s10198-020-01197-6
45. Rice DB, Carboni-Jimenez A, Canedo-Ayala M, et al. Perceived Benefits and Faciliatators and Barriers to Providing Psychosocial Interventions for Informal Caregivers of People with Rare Diseases: A Scoping Review [Review]. *The Patient: Patient-Centered Outcomes Research* 2020;13(5):471-519.
46. Landfeldt E, Lindgren P, Bell CF, et al. Quantifying the burden of caregiving in Duchenne muscular dystrophy. *Journal of Neurology* 2016;263(5):906-15. doi: 10.1007/s00415-016-8080-9
47. Selman LE, Beynon T, Radcliffe E, et al. 'We're all carrying a burden that we're not sharing': a qualitative study of the impact of cutaneous T-cell lymphoma on the family. *British Journal of Dermatology* 2015;172(6):1581-92. doi: 10.1111/bjd.13583
48. Sloper T, Beresford B. Families with disabled children *BMJ* 2006;333(7575):928-29.
49. Hanbury A, Smith A.B., K B. Deriving Vignettes for the Rare Disease AADC Deficiency Using Parent, Caregiver and Clinician Interviews to Evaluate the Impact on Health-Related Quality of Life. *Patient Related Outcome Measures* 2021;12:1-12.
50. Currie G, Szabo J. “It is like a jungle gym, and everything is under construction”: The parent's perspective of caring for a child with a rare disease. . *Child: Care, Health and Development* 2019;45(1):96-103.
51. Currie G, Szabo J. 'It would be much easier if we were just quiet and disappeared': Parents silenced in the experience of caring for children with rare diseases. *Health Expectations* 2019;22(6):1251-59.
52. Mutch K, Methley A, Hamid S, et al. If they are OK, we are OK: The experience of partners living with neuromyelitis optica. *Disability and Rehabilitation* 2017;39(13):1279-86.

For peer review only

53. McMullan J, Crowe A, Bailie C, et al. Improvements needed to support people living and working with a rare disease in Northern Ireland: current rare disease support perceived as inadequate. *Orphanet J Rare Dis* 2020;15(315)

For peer review only

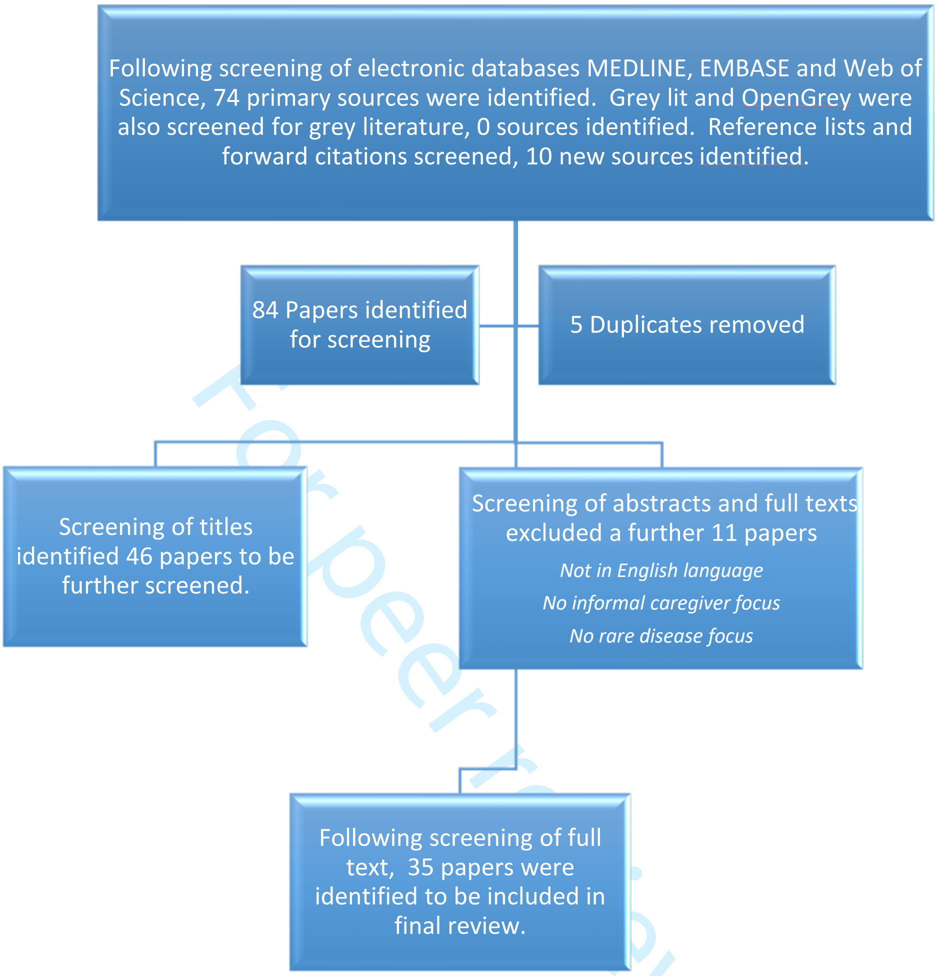

Figure 1 PRISMA flowchart

For peer review only

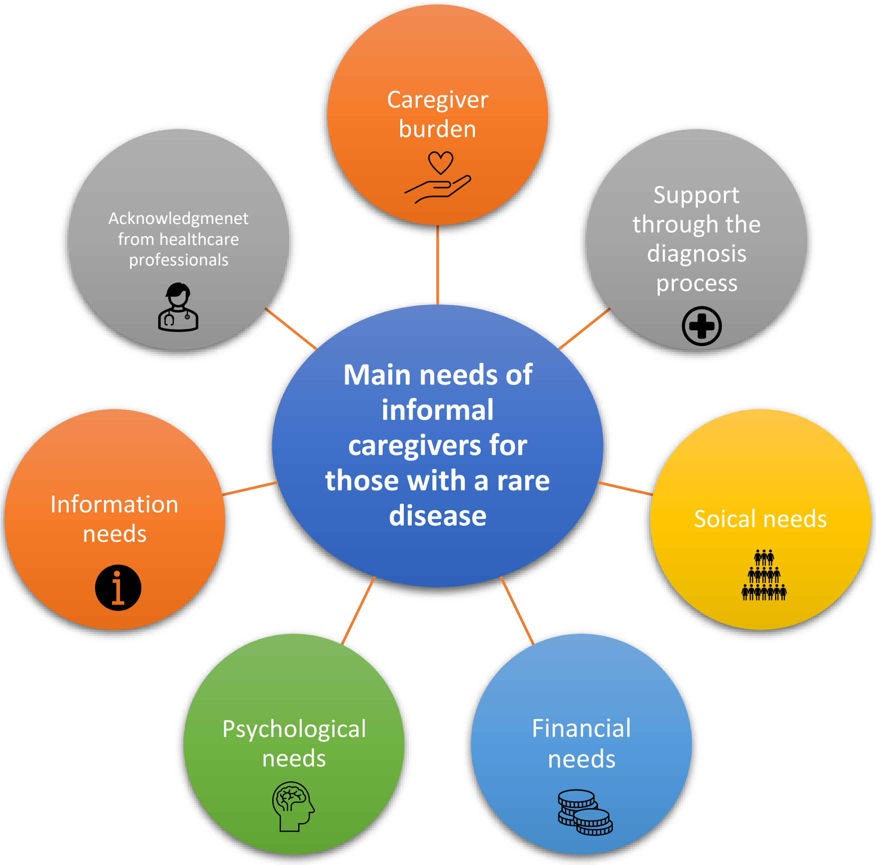

Figure 2 Main needs of caregivers for those with a rare disease

For peer review only

Supplementary file 1: Data extraction table				
**Author & year of publication**	**Country in which the study was conducted**	**Data collection method(s)**	**Participants**	**Identified caregiver needs**
Anderson, M., Elliott EJ, Zurynski YA.<br><br>2009	Australia	Survey	Parents/carers	• Support around diagnosis.<br>• Access to peer support groups.<br>• Psychological support.
Applebaum A.J., Polacek L.C., Walsh L. *et al.*<br><br>2020	USA	Survey and semi-structured interview	Caregivers of patients with Erdheim-Chester disease	• Social connections to prevent extreme isolation
Aubeeluck A.V. Buchanan H.E., Stupple E.J.N.<br><br>2011	England	Focus groups	Caregivers of those with Huntington's Disease	• More time to focus on themselves.
Craig T.J., Banjerji A., Riedl M.A., *et al.*<br><br>2021	USA	Online survey	Adults caring for an individual with Hereditary angioedema (HAE)	• Recognition for the important role they have.<br>• Inclusion in treatment discussions.
Hanbury A, Smith AB & Buesch K<br><br>2021	England	Vignettes were developed	Caregivers	• Engagement with research
Baumbusch, J., Mayer, S., & Sloan-Yip, I.<br><br>2018	Canada	Semi-structured interviews	Parents of children with a RD	• Acknowledgment from healthcare providers<br>• Support coordinating care<br>• Peer support – information and emotional<br>• Policies and programs to validate their role<br>• Support from genetic counsellor to connect them to others

For peer review only

Bendixen, R. M., & Houtrow, A.<br><br>2017	USA	Interviews	Parents of children with Duchenne muscular dystrophy	• Listened to by healthcare providers<br>• Inclusion in the diagnostic process<br>• Improved delivery of the diagnosis<br>• Improved follow up after diagnosis
Cañedo-Ayala, M., Rice, D. B., Levis, B., et al.<br><br>2020	USA	Questionnaire	Informal caregivers	• Mental health support
Currie, G., & Szabo, J.<br><br>2019	Canada	Semi-structured interviews	Parents of children with RDs	• Communication issues need addressed as parents often know more about the disease than healthcare providers.<br>• Improved coordination of care between providers and services caring for children with rare diseases.<br>• Gap in accessibility to government supports needs addressed.
Currie, G., & Szabo, J.<br><br>2019	Canada	Semi-structured interviews	Parents of children with rare neurodevelopmental diseases	• Health-care providers need more understanding of rare conditions.<br>• More support needed, parents often 'lose themselves' in this demanding caring role.
Flores D., Ribate M.P., Montolio M., *et al.*<br><br>2020	Spain	Questionnaires	Caregivers to patients with Duchenne muscular dystrophy	• Financial support.
Kanters T.A., van der Ploeg Ans. T, Brouwer W.B.F., *et al.*<br><br>2013	Netherlands	Questionnaire	Caregivers for those with Pompe disease.	• Support needed for informal caregivers.
Kasparian, N. A., Rutstein, A., Sansom-Daly, *et al.*	Australia	Telephone interviews	Patients and caregivers affected by Von Hippel-Lindau disease	• More supportive care services requested.

For peer review only

2015				
Landfelt E., Lindgren P., Bell C.F., *et al.*<br><br>2016	Germany, Italy, UK, USA	Visual analogue scale, survey, interview	Caregivers to patients with Duchenne muscular dystrophy	• Screening for depression.<br>• Holistic approach to family mental health needed.
Lagae, L., Irwin, J., Gibson, E., *et al.*<br><br>2019	Europe	Survey	Caregivers for those with Dravet syndrome (DS)	• Time needed to escape caring duties.<br>• Formal support and respite required.<br>• Stress is common – support needed.
Lopez-Bastida J., Pena-Longobardo L.M., Aranda-Reneo I, *et al.*<br><br>2017	Spain	Questionnaire	Patients and caregivers with Spinal Muscular Atrophy	• Financial support<br>• QoL must be addressed
Lyon, M. E., Thompkins, J. D., Fratantoni, K., *et al.*<br><br>2019	USA	Semi-structured interviews	Caregiving families	• Worries about the future need to be addressed.<br>• More time for themselves needed.<br>• Financial concerns must be addressed.
McKnight, A. J. M., Walker, R., Collins, C. (2020).	Northern Ireland	Report	N/A	• Access to accurate information<br>• Access to appropriate services<br>• Improved communication
McMullan, J., Crowe, A. L., Bailie, C. *et al.*<br><br>2020	Northern Ireland	Survey and semi-structured interviews	Rare disease collaborative groups	• Caregivers often overlooked in RD research – their opinions and experiences must be valued.
McMullan J, Crowe A.L., Downes K., *et al.*<br><br>2021	Northern Ireland	Survey and workshop	Caregivers of those with a rare disease	• Improved interactions with healthcare professionals.<br>• Improved emotional, psychological and social support.<br>• Assistance with finances.<br>• Better awareness of support services.
Mooney J., Graham K. & Watts R.A.	England	Semi-structured interviews	Patients with ANCA-associated vasculitis and their caregivers.	• Emotional support is needed.<br>• Reassurance about the future.

For peer review only

2019				
Mori, Y., Downs, J., Wong, K., *et al.*<br><br>2017	Australia	Survey	Families with a child with CDKL5 disorder	• Burden of daily caregiving requires support, particularly in relation to emotional wellbeing, sleep problems, financial difficulties, QoL.
Mutch, K., Methley, A., Hamid, S., *et al.*<br><br>2017	England	Semi-structured interviews	Partners of people with NMO	• Acknowledgement from HCP regarding the vital role they play in caring.
Palacios-Cena, D., Famoso-Perez, P., Salom-Moreno, J., *et al.*<br><br>2018	Spain	Interviews, focus groups, researcher's field notes, caregiver's personal documents	Caregivers of children with Rett Syndrome	• Answers needed regarding 'the first symptoms' and 'the need for a diagnosis'.<br>• Help with managing day to day life.<br>• Financial support.
Pelentsov, L. J., Fielder, A. L., & Esterman, A. J.<br><br>2016	Australia	Semi-structured focus group interviews	Parents of a child with a RD	• Social isolation must be addressed.<br>• Knowledge of HCP needs to be improved.<br>• Support needed as family relationship often impacted due to demands of caring.
Pelentsov, L. J., Laws, T. A., & Esterman, A. J.<br><br>2015	Australia	Scoping Review	Parents of child with a RD	• Improved parental supportive care – common unmet needs between RDs.
Rice D.B., Canedo-Ayala M., Carboni-Jimeniez A., Carrier M-E, *et al.*<br><br>2020	USA	Online questionnaire	Caregivers of people with SSc	• Emotional support is required.<br>• Help with physical needs.<br>• Interventions needed delivered through hardcopy or online resources, including those delivered after the care recipient's diagnosis.
Rice D.B., Carbino-Jimeniz A., Canedo-Ayala M. *et al.*<br><br>2020	USA	Scoping review	N/A	• Psychosocial interventions needed to reduce caregivers stress, burden and feelings of isolation among caregivers.

For peer review only

				• Future research should design interventions for caregivers.
Rodriguez A.A., Martinez O., Amayra I, *et al.*<br><br>2021	Spain	Questionnaire	Carers of children with neuromuscular disease	• Financial assistance.<br>• Employment support.<br>• Physical and psychological support.
Selman L.E., Beynon T., Radcliffe S., *et al.*<br><br>2014	London	Semi-structured qualitative interviews	Adult informal caregivers of patients with Cutaneous T-cell lymphoma	• Easily accessible services are needed that include the family in the unit of care, provide support and information, and understand the process of family adjustment and adaptation.
Sloper, T., & Beresford, B.<br><br>2006	UK	Report	Families of disabled children	• Policies and structures to support social and economic needs.
Somanadhan, S., & Larkin, P. J.<br><br>2016	Ireland	In-depth interviews	Parents of those with Mucopolysaccharidosis	• Reassurance and certainty needed about the future.
Wiblin, L., Durcan, R., Lee, M., *et al.*<br><br>2017	England	Qualitative in depth interviews	Patients and caregivers living with MSA and PSP	• Better connections to others to reduce social isolation.<br>• Improved communication.
Williams, J. K., Skirton, H., Paulsen, J. S., *et al.*<br><br>2009	USA, Canada	Focus groups	Adult caregivers of people with Huntington's disease	• Emotional distress support should be provided.<br>• Assistance in managing several roles.<br>• Mental health monitoring.
Wu, Y., Al-Janabi, H., Mallett, A., *et al.*<br><br>2020	Australia	Clinical data was used from Mitochondrial Disease, Epileptic	Parents of those with rare genetic conditions	• Health effects on family members need to be considered.

For peer review only

		Encephalopathy and Brain Malformation projects		

For peer review only

BMJ Open

The needs of informal caregivers of people with a rare disease: a rapid review of the literature.

Journal:	*BMJ Open*
Manuscript ID	bmjopen-2022-063263.R1
Article Type:	Original research
Date Submitted by the Author:	15-Sep-2022
Complete List of Authors:	Mcmullan, Julie; Queen's University Belfast Faculty of Medicine Health and Life Sciences, Centre for Public Health<br>Lohfeld, Lynne; Wenzhou Medical University Eye Hospital, ; Queen's University Belfast, Centre for Public Health<br>McKnight, Amy; Queen's University Belfast, Centre for Public Health
**Primary Subject Heading**:	Public health
Secondary Subject Heading:	Public health
Keywords:	Quality in health care < HEALTH SERVICES ADMINISTRATION & MANAGEMENT, Health policy < HEALTH SERVICES ADMINISTRATION & MANAGEMENT, PUBLIC HEALTH

SCHOLARONE™
Manuscripts

I, the Submitting Author has the right to grant and does grant on behalf of all authors of the Work (as defined in the below author licence), an exclusive licence and/or a non-exclusive licence for contributions from authors who are: i) UK Crown employees; ii) where BMJ has agreed a CC-BY licence shall apply, and/or iii) in accordance with the terms applicable for US Federal Government officers or employees acting as part of their official duties; on a worldwide, perpetual, irrevocable, royalty-free basis to BMJ Publishing Group Ltd ("BMJ") its licensees and where the relevant Journal is co-owned by BMJ to the co-owners of the Journal, to publish the Work in this journal and any other BMJ products and to exploit all rights, as set out in our licence.

The Submitting Author accepts and understands that any supply made under these terms is made by BMJ to the Submitting Author unless you are acting as an employee on behalf of your employer or a postgraduate student of an affiliated institution which is paying any applicable article publishing charge ("APC") for Open Access articles. Where the Submitting Author wishes to make the Work available on an Open Access basis (and intends to pay the relevant APC), the terms of reuse of such Open Access shall be governed by a Creative Commons licence – details of these licences and which Creative Commons licence will apply to this Work are set out in our licence referred to above.

Other than as permitted in any relevant BMJ Author's Self Archiving Policies, I confirm this Work has not been accepted for publication elsewhere, is not being considered for publication elsewhere and does not duplicate material already published. I confirm all authors consent to publication of this Work and authorise the granting of this licence.

For peer review only

1

The needs of informal caregivers of people with a rare disease: a rapid review of the literature.

Authors

Julie McMullan (PDRF) [1], Lynne Lohfeld (Lecturer) [1], Amy Jayne McKnight (Professor) [1]

Institutional address

[1] Centre for Public Health, School of Medicine Dentistry and Biomedical Sciences, Institute of Clinical Science Block A, Grosvenor Road, Belfast, BT12 6BA.

Corresponding author

Julie McMullan

Email: raredisease@qub.ac.uk

Abstract

Objectives: Many people living with a rare disease are cared for by a family member. Due to a frequent lack of individual rare disease (RD) knowledge from healthcare professionals, the patient and their informal caregiver are frequently obliged to become 'experts' in their specific condition. This puts huge strain on family life and results in caregivers juggling multiple roles in addition to unique caring roles including as advocate, case manager, and medical navigator. We conducted a rapid review of literature reporting on the unmet needs of informal caregivers for people living with a RD.

Setting: Searches were conducted in Medline, Embase, Web of Science, GreyLit and OpenGrey.

Results: Thirty-five papers were included in the final review & data extracted. This rapid review presents several unmet needs identified by informal caregivers of persons with a RD. The related literature was organised thematically: caregiver burden, support through the diagnosis process, social needs, financial needs, psychological needs, information and communication needs and acknowledgement from healthcare professionals.

Conclusions: This review provides evidence that increased meaningful support is required for caregivers. Active engagement should be encouraged from this cohort in future research and awareness raised of the support available to improving the quality of life for families living with an RD.

For peer review only - http://bmjopen.bmj.com/site/about/guidelines.xhtml

The unmet needs identified through this review will benefit people living with a RD, caregivers, healthcare professionals and policy makers.

Key words: burden, informal caregiver, needs, rare disease, review.

Strengths and limitations

- This study explored a cohort of individuals who frequently go unnoticed and unreported in the literature – informal caregivers of individuals diagnosed with a rare disease.
- The rapid review followed the guidelines for conducting a rapid review of the literature.
- The review was conducted by experienced researchers in the area of RD carer needs who have a wealth of relevant experience. Additionally, manual searches of reference lists were conducted to identify any papers not found by the initial search strategy.
- It is important to acknowledge limitations of this review. Due to resource constraints, only one author (JM) initially screened the titles and abstracts from the total set of documents retrieved. This could lead to bias.
- Conducting a rapid review produced results quickly, including identifying the main self-identified needs of caregivers of people living with an RD. This is particularly important given the key role that informal caregivers play, and the fact that earlier research has focused on the person with an RD and not the caregiver.

The authors of this paper consider the needs of RD caregivers a priority and recognises the value they bring to the unique caring needs.

Introduction

In Europe, rare diseases (RDs) are those which affect less than 1 in 2000 people in a specified population [1]. Although each RD occurs infrequently, collectively RDs are a major public health issue affecting more than 450 million people globally [2 3]. RDs result in a wide variety of healthcare needs stemming from the involvement of multiple organ systems, such as respiratory complications, the circulatory system. Muscular system, digestive system, and central nervous system. Many RDs are chronic, complex and associated with physical, intellectual, or neurological disabilities that significantly affect patients and their families. In addition, many families living with a RD lack peer and community support services [4-7]

For peer review only

3

Although not always the case, family often plays a pivotal role in a person's adjustment to chronic disease and is influenced by behavioural and social factors [8]. A family member frequently takes on the role as caregiver or a person with a close relationship as an informal caregiver. Caring for someone with an RD affects many areas of life including psychologically, economically, physically and logistically [9]. The importance of good mental and physical health for the informal caregiver is therefore vital for their own wellbeing and to ensure they can sustain the essential role of assisting the person with an RD [10 11]. Individuals with RDs and their families often have limited evidence-based information to guide decisions about disease management and symptom relief [12 13]. Further, the inherent uncertainty that comes with having a RD, including delays in diagnosis (the average time for a diagnosis of a rare disease in Northern Ireland is 5 years) and a lack of knowledge about current and future care needs [14 15], impact access to services and management of the RD [16]. Research on the experience of having an RD indicates that care and service needs are often not identified based on the severity of the health condition, but rather are associated with poor quality of care and barriers to access, for example lower satisfaction with healthcare services and care coordination [15 17].

Given the few people with a specific RD, healthcare providers often are not knowledgeable about the condition. Therefore people living with a RD and their carers have to become their own experts [18]. This causes a change to the usual patient-doctor relationship, which can bring challenges such as difficulties in communication and patients struggling to get sufficient accurate information to make informed choices [19 20].

Caring for someone with a RD can be highly demanding often requiring intense and unique care specific to the individuals needs [21]. Delayed diagnosis, lifelong caring, limited capacity for independent living, lack of treatment options and large health service needs have severe impacts on parent's physical and psychosocial wellbeing [22].

Rapid reviews are an emerging type of knowledge synthesis used to inform health-related policy decisions and discussions, especially when information needs are immediate [23-26]. Rapid reviews streamline systematic review methods – for example, by focusing the literature search [23] while still aiming to produce valid conclusions. The requirements or the review, which was undertaken with a short deadline, were for a short but in-depth synthesis of the current state of the issues facing caregivers for those with a rare disease.

For peer review only

This review focuses on informal caregivers for people living with a RD. We were particularly interested in their holistic needs when caring for this unique population and how these may differ from other caring populations where there is often more support available e.g. carers of people with dementia or cancer.

Methodology

Search strategy and inclusion criteria

Three electronic databases were searched -- Medline, Embase and Web of Science -- using the combined terms 'informal caregiver*' and 'rare disease*'. All searches were conducted on 14 September 2021, with no date restrictions. Reference lists of included papers were screened for further sources. A search was also conducted of grey literature using the databases GreyLit and OpenGrey. Duplicates and non-English language articles were excluded. The criteria for inclusion were articles that address caregiving for people living with an RD. Articles on caregiving alone or RD alone were excluded. Primary research studies and systematic review were considered for inclusion as shown in Table 1.

Study selection and data extraction

Database searches were last conducted on 14 September 2021 by JM. Titles and abstracts of the identified articles were downloaded onto Endnote. Duplicate articles were removed and the remaining papers were screened through analysing their titles and abstracts. Additionally, manual searches of reference lists were conducted on 21 September 2021 to identify any papers not found by the initial search strategy. If relevant, the papers were then further screened by reading the full text. Data were extracted by JM who recorded the follow data for each study:

- Author & year of publication
- Country in which the study was conducted
- Data collection method(s)
- Participants
- Identified caregiver needs

The identified caregiver needs were extracted from the included papers [JM] and presented the data extraction table [AMcK, JM & LL] (Supplementary file 1). From this, common needs were grouped

together under headings which became the themes for this review. The themes and content for the themes was agreed between the research team [AMcK, JM & LL].

Table 1: Literature search results

Database	No. of articles retrieved	No. of articles included in review
Medline	6	5
Embase	6	5
Web of Science	62	13
GreyLit	0	0
OpenGrey	0	0

This table shows the number of articles identified from each database before reference lists were checked.

An illustration of the search strategy including databases searched and screening methods is displayed in Figure 1, modelled on the PRISMA flow diagram.

Patient and Public Involvement

The need for this review was highlighted by a priorities workshop in 2020, which considered contributions from >2,000 individuals living with rare disease. The review was also shared with representatives from a local rare disease charity in Northern Ireland (the Northern Ireland Rare Disease Partnership).

Results

General description of the literature

Sources initially identified from each database were as follows: MEDLINE *n= 6*, Embase *n= 6*, Web of Science *n= 62*, OpenGrey *n=0* and GreyLit *n=0*. The screening process resulted in 30 documents which were reviewed based on their titles and abstracts. Following the screening 5 duplicate papers were removed and 25 papers were identified for full text screening. Further studies were identified from searching the reference lists. Finally, 35 texts were included in the full review, with characteristics of each source summarised from the completed data extraction table (Supplementary file 1).

Of the texts included, publication dates were from 2006 to 2021 and the studies were conducted in Australia, Canada, Germany, Ireland, Italy, Netherlands, Spain, United Kingdom and the United States. The identified studies focus on many rare diseases including Hereditary angioedema, Huntington's Disease, Duchenne muscular dystrophy and Von Hippel-Lindau disease.

A narrative review is presented below, highlighting the main needs of caregivers of people living with an RD. The related literature has been organised thematically under the following headings:

- Caregiver burden
- Support through the diagnosis process
- Social needs
- Financial needs
- Psychological needs
- Information and communication needs
- Acknowledgement from healthcare professionals

Findings from these studies were organised thematically under the following headings as shown visually in Figure 2.

Caregiver burden

Caring for someone with an RD presents a range of challenges [27 28] that are affected by the severity of the illness and its duration, knowledge about the condition and its changes over time, and one's ability to address the emotional toll involved with long-term care [29]. Despite the stress and difficulties associated with being a family caregiver [30], often they acknowledge the positive aspects of caring for their loved ones [30].

The quality of life of caregivers is often compromised by their placing others' needs before their own [31]. Previous research has shown that informal caregivers do not have enough time for themselves, [32] and that caring for someone with an RD can negatively impact on all dimensions of family life [33]. Caregivers are often forced to miss work or school days due to the demands of their role, frequently address unpleasant events and watch a loved one suffer [28]. Consequently, they would benefit greatly from support to reduce the burden of caring for example from support groups, respite care and employment arrangements [34]. This includes both supports for informal carers of people with a variety of health issues, as well as for RD-related issues [34].

Support through the diagnosis process

For peer review only

7

In spite of marked medical progress over the past several decades in identifying an RD, there are substantial delays in diagnosing these conditions [35]. This can have adverse impacts on families, for example, financial and mental health [12]. Little attention is given to parent concern in the diagnostic process with previous research reporting concerns regarding how the diagnosis was delivered to parents, lack of guidance and poor follow-up post diagnosis [35]. Previous research has shown that families are often dissatisfied by the way in which a diagnosis is given, including an insensitive style of communication, not offering support or counselling, and inadequate provision of information about the disease. This can cause emotional stress for caregivers who have not yet learned about the condition and how it will affect them and their families. Therefore, interventions to support the following a diagnosis would be useful, delivered either in hardcopy or online [36].

Social needs

Social isolation is common among caregivers with the impact on personal relationships being a reoccurring theme [4 5]. All too often, caregivers have little time to themselves and lack appropriate support and time for respite. This in turn causes further emotional stress and in the case of parents, uncertainty about their child's future [37]. Parents of children with an RD often feel isolated from mainstream society and struggle to stay socially connected [4]. It was suggested that the insights gained through research studies regarding the impact on caregivers' social lives should be considered in future clinical service planning. Additionally holistic, empathic, and person-centred medical and psychosocial care is urgently needed for this cohort [6]. Support is needed as relationships among family members are often impacted due to demands of caring [4]. There is a need for improved parental supportive care as many common unmet needs exist across RDs [38].

Financial needs

Previous research has indicated that financial issues are a top concern of caregivers of persons with RD [30 6 39 27]. The high economic costs that families must cover means that financial burden is common among caregivers of those with an RD, many of whom are forced to exhaust their financial savings [40]. Purchasing equipment, hiring professionals, and the additional financial burden the illness has on families poses immense stress on family life [41]. Often financial issues impact on the ability of the caregiver to meet the individual's healthcare needs, increasing the strain on their lives [42]. Substantial social/economic burden is mostly attributable to high direct non-healthcare costs [43]. Previous research has shown that caregivers' financial burden might be conditioned by the clinical condition of the patient [44]. In addition to the direct healthcare costs, caregivers of those with an RD report that their career choices are influenced due to their caring role. Furthermore, caring duties can result in

missed working days due to emergency caring demands [37] making it difficult for many caregivers to maintain their job and advance in their careers [41]. Financial hardship adversely effects the mental health of caregivers [42].

Psychological needs

There is a lack of psychological support for families caring for children with a RD, and many report that accessing appropriate psychological care is difficult [6 12]. Previous research has suggested that this form of support should be offered at the time of diagnosis [12 10]. Depressive symptoms are often associated with caregiving burden and therefore there is a need to develop interventions in additional to promoting the existing validated tools for caregivers considering their special needs [10]. Caregivers often express concerns about the future [32], experiencing emotional distress that can compromise the well-being of family carers, who attempt to maintain multiple roles [11]. Psychological interventions can help reduce stress, a sense of being burdened and feelings of isolation that many RD caregivers feel [45]. Research has suggested that screening for depression is needed and emphasises the need for a holistic approach to family mental health in the context of chronic childhood disease [46]. Peer support is a key resource in terms of information and emotional support for parents who often begin their journey feeling isolated and alone [21].

Information and communication needs

Suggestions were made that information about the condition should be given to caregivers at the time of diagnosis as well as signposting them to groups connecting families who share common experiences [21]. Caregivers require access to accurate information, appropriate services and improved communication between patients, families, and a range of both health and social care and other public services [2]. Families require easily accessible services that include the family in the unit of care, provide support and information, and understand the process of family adjustment and adaptation in the long term [47]. Policies and structures are needed to support social and economic needs [48]. Engaging caregivers in future avenues of research is vital to ensure resources and funding are targeted in the best way [49].

Acknowledgement from healthcare professionals

Out of necessity, informal carers often know more about a particular RD than healthcare providers do. This often leads to poor communication and collaboration. Furthermore a lack of coordination of care force carers to fill the gap by juggling multiple roles including that of advocate, case manager, and medical navigator [50]. Caregivers often experience silencing or being silenced when interacting

For peer review only

with health-care and social care systems and providers [51]. This has been attributed to the lack of knowledge about RDs by health-care providers who also are unaware of the impact that caring for someone with RD [50]. This points to the need to improve the knowledge of health care providers on the medical, social and financial impact these informal carers experience [4]. Health care providers should also acknowledge the vital role that informal caregivers play in promoting the health and quality of life of persons with an RD [52]. Caregivers, especially parents caring for someone with an RD, work hard to be heard and acquire services within health and social systems [50]. Another way that informal caregivers are silenced is due to how they are overlooked in the world of research despite growing emphasis on ensuring 'patient and public involvement'. It is vital that this pattern is changed so that they have an opportunity to be heard [53 27].

Discussion

The purpose of this rapid review was to synthesise and describe what is currently known about the needs of informal caregivers for people living with an RD. Based on the findings, several recommendations for future healthcare practices and policies, as well as for research are evident. First, it is important to consider the extreme burden often experienced by these caregivers, many of whom place the need of others ahead of their own while navigating this journey with little or no support [31]. Support is needed to help caregivers with many aspects of their caring duties from the initial diagnosis process which has been a difficult time for many families [35].

Social support would be welcomed to enable caregivers to have much needed time away from their caring duties to recharge and also to relieve them of the emotional strain which impacts on not only themselves but their wider family [4]. The mental strain of caregiving is evident from previous research, psychological interventions are required that consider the family as a whole to reduce the emotional strain and depression too often experienced by caregivers [21 45]. Many caregivers have also reported struggling financially not only due to the high costs of the practical side of caring but as a result of the impact caring can have on their ability to maintain a career [41], financial support therefore a priority.

Clear information should be provided for caregivers from diagnosis through to their rare disease journey [2] to replace the current situation of confusion and uncertainty due to unclear and incomplete or conflicting messages. Lastly, caregivers must be recognised by healthcare professionals for the integral part they have in caring for someone with a rare disease. This relationship often changes the dynamic between health professional and patient and so caregivers must be listened to and viewed

For peer review only

as an 'expert'[52]. All of the above-mentioned issues should be taken into consideration when planning future research, it is vital that informal caregivers views are valued.

Conclusion

This rapid review presents several unmet needs identified by informal caregivers of persons with a RD. It is hoped that the findings contribute to increasing the amount of meaningful support for caregivers as well as encourage their active participation in improving the quality of life for families living with an RD. It is important that this cohort are engaged in future research to ensure their needs are being addressed. The insights that are gained through future research in relation to the impact on caregivers' social lives should be considered in the priorities and strategic directions for clinical services. Interventions to support them following a diagnosis would be useful, delivered either in hardcopy or online. Better meeting needs of informal caregivers for people living with a RD is in the best interests of people with a RD, healthcare professionals and policy makers, as well as caregivers. It is important that awareness is raised about the range of support options are available for informal caregivers of people with an RD from health and social care providers, charities and/or support groups [27].

Abbreviations

RD Rare disease

Declarations

- Ethics approval and consent to participate: Not applicable
- Consent for publication: Not applicable
- Availability of data and materials: Not applicable
- Competing interests: The authors declare that they have no competing interests.
- Funding: JM was supported by an award from the NI Public Health Agency and the Medical Research Council (MC_PC_16018) – Northern Ireland Executive support of the Northern Ireland Genomic Medicine Centre though Belfast Health and Social Care Trust.
- Authors' contributions: JM contributed to data curation, formal analysis, data interpretation, drafting and reviewing the original manuscript. LL & AJM contributed to study conception and design, data interpretation, project administration, supervision and reviewing manuscript drafts. All authors approved the final version of the manuscript for submission.
- Acknowledgements: Not applicable

For peer review only - http://bmjopen.bmj.com/site/about/guidelines.xhtml

Reference list

1. Beaulieu CL, Majewski J, Schwartzentruber J, et al. FORGE Canada Consortium: outcomes of a 2-year national rare-disease gene discovery project. *American Journal of Human Genetics* 2014;94(6):809-17.
2. McKnight A, Walker R, Collins C. Communications and Information Review: NI Report for Rare Diseases, 2020.
3. McMullan J, Crowe A, Kerr K, et al. Perspectives on a NI Rare Disease Registry, 2020.
4. Pelentsov LJ, Fielder AL, Esterman AJ. The supportive care needs of parents with a child with a rare disease: A qualitative descriptive study. *Journal of Pediatric Nursing* 2016;31(3):e207-e18.
5. Wiblin L, Durcan R, Lee M, et al. The importance of connection to others in QoL in MSA and PSP. *Parkinsons Disease* 2017:1-9.
6. Kasparian NA, Rutstein A, Sansom-Daly UM, et al. Through the looking glass: An exploratory study of the lived experiences and unmet needs of families affected by Von Hippel-Lindau disease. *European Journal of Human Genetics* 2015;23(1):34-40.
7. Kole A, Faurisson F. The Voice of 12,000 Patients: Experiences and expectations of rare disease patients on diagnosis and care in Europe. Eurordis2009 [Available from: http://www.eurordis.org/publication/voice-12000-patients accessed 9 September 2021.
8. Buchanan H, Huang C. Health-related quality of life among informal caregivers assisting people with multiple sclerosis. *Disability and Rehabilitation* 2011;33(2):113-21.
9. Mulroy S, Robertson L, Aiberti K, et al. The impact of having a sibling with an intellectual disability: Parental perspectives in two disorders. *J Intellect Disabil Res* 2008;52:216-29.
10. Canedo-Ayala M, Rice DB, Levis B, et al. Factors associated with symptoms of depression among informal caregivers of people with systemic sclerosis: A cross-sectional study. *Disability and Rehabilitation* 2020;42(3):394-99.
11. Williams JK, Skirton H, Paulsen JS, et al. The emotional experiences of family carers in Huntington disease. *Journal of Advanced Nursing* 2009;65(4):789-98.
12. Anderson M, Elliott EJ, Zurynski YA. Australian families living with rare disease: experiences of diagnosis, health services use and needs for psychosocial support. *Orphanet J Rare Dis* 2013;8:22. doi: 10.1186/1750-1172-8-22 [published Online First: 2013/02/13]
13. Forsythe LP, Szydiowski V, Murad MH, et al. A systematic review of approaches for engaging patients for research on rare diseases. *Journal of General Internal Medicine* 2014;29(53):788-800.
14. Grut L, Kvam MH. Facing ignorance: people with rare disorders and their experiences with public health and welfare services. *Scandinavian Journal of Disability Research* 2013;15(1):20-32.
15. Huyard C. What if anything is specific about having a rare disorder? Patients' judgements on being ill and being rare. *Health Expectations* 2009;12(4)
16. Delive L, Samuelsson L, Tallborn A, et al. Stress and well-being among parents of children with rare diseases: a prospective intervention study. *Journal of Advanced Nursing* 2006;53(4):392-402.
17. Farmer JE, Marien WE, Clark MJ, et al. Primary care supports for children with chronic health conditions: identifying and predicting unmet family needs. *Journal of Pediatric Psychology* 2004;29(5):355-67.
18. Crowe A, McKnight A, McAneney H. Communication needs for individuals with rare diseases within and around the healthcare system of Northern Ireland. *Frontiers in Public Health* 2019;7(236)
19. Budych K, Helms TM, C. S. How do patients with rare diseases experience the medical encounter? Exploring the role behavior and its impact on patient-physician interaction. *Health Policy* 2012;105(2-3):154-64.

For peer review only

20. Dudding-Byth T. A powerful team: The family physican advocating for patients with a rare diease. *Australian Family Physician* 2015;44(9):634-38.
21. Baumbusch J, Mayer S, Sloan-Yip I. Alone in a crowd? Parents of children with rare diseases' experiences of navigating the healthcare system. *Journal of Genetic Counseling* 2018;28(1):80-90.
22. Madeo AC, K.E. OB, Bernhardt BA, et al. Factors associated with perceived uncertainty among parents of children with undiagnosed medical conditions. *American Journal of Medical Genetics* 2012;158A(8):1887-84.
23. Watt A, Cameron A, Sturm L, et al. Rapid reviews verus full systematic reviews: an inventory of current methods and practice in health technology assessment. . *International Journal of Technology Assessment in Health Care* 2008;24:133-39.
24. Ganann B, Ciliska D, Thomas H. Expediting systematic reviews: methods and implications of rapid reviews. . *Implementation Science* 2010;5(56)
25. Khangura S, Konnyu K, Cushman R, et al. Evidence summaries: the evolution of a rapid review approach. *Systematic reviews* 2012;1(10)
26. Konnya K, Kwok E, Skidmore B, et al. The effectiveness and safety of emergency department short stay units: a rapid review. *Open Medicine* 2012;6(e10-e16)
27. McMullan J, Crowe A, Downes K, et al. Carer reported experiences: Supporting someone with a rare disease. *Health and Social Care in the Community* 2021;0:1-12.
28. Craig TJ, Banerji A, Riedl MA, et al. Caregivers' role in managing hereditary angioedema and perceptions of treatment-related burden. *Allergy and Asthma Proceedings* 2021;42:S11-S16. doi: 10.2500/aap.2021.42.210029
29. Mooney J, Graham K, Watts RA. Impact of caring for someone with a rare rheumatic condition, views from patients and informal carers-the need for cat-like vigilance. *Rheumatology Advances in Practice* 2019;3(1) doi: 10.1093/rap/rkz003
30. Kanters TA, van der Ploeg AT, Brouwer WBF, et al. The impact of informal care for patients with Pompe disease: An application of the CarerQol instrument. *Molecular Genetics and Metabolism* 2013;110(3):281-86. doi: 10.1016/j.ymgme.2013.07.020
31. Aubeeluck AV, Buchanan H, Stupple EJN. 'All the burden on all the carers': Exploring quality of life with family caregivers of Huntington's disease patients. *Quality of Life Research* 2012;21(8):1425-35.
32. Lyon ME, Thompkins JD, Fratantoni K, et al. Family caregivers of children and adolescents with rare diseases: A novel palliative care intervention. *BMJ Supportive and Palliative Care* 2019;25(25)
33. Somanadhan S, Larkin PJ. Parents' experiences of living with, and caring for children, adolescents and young adults with Mucopolysaccharidosis (MPS). . *Orphanet J Rare Dis* 2016;11(1):138.
34. Wu Y, Sun F, Lee P. Family caregivers' lived experiences of caring for epidermolysis bullosa patients: A phenomenological study. *Journal of Clincial Nursing* 2020;29(9-10):1552-60.
35. Bendixen RM, Houtrow A. Parental reflections on the diagnostic process for duchenne muscular dystrophy: A qualitative study. *Journal of Pediatric Health Care* 2017;31(3):285-92.
36. Rice DB, Canedo-Ayala M, Carboni-Jimenez A, et al. Challenges and support service preferences of informal caregivers of people with systemic sclerosis: a cross-sectional survey. *Disability & Rehabiliation* 2020;42(16):2304-10.
37. Lagae L, Irwin J, Gibson E, et al. Caregiver impact and health service use in high and low severity Dravet syndrome: A multinational cohort study. *Seizure* 2019;65:72-77.
38. Pelentsov LJ, Laws TA, Esterman AJ. The supportive care needs of parents caring for a child with a rare disease: A scoping review. *Disability and Health Journal* 2015;8(4):475-91.
39. Rodriguez AA, Martinez O, Amayra I, et al. Diseases Costs and Impact of the Caring Role on Informal Carers of Children with Neuromuscular Disease. *International Journal of Environmental Research and Public Health* 2021;18(6) doi: 10.3390/ijerph18062991

For peer review only

40. Applebaum AJ, Polacek LC, Walsh L, et al. The unique burden of rare cancer caregiving: caregivers of patients with Erdheim-Chester disease. *Leukemia Lymphoma* 2020;61(6):1406-17.
41. Palacios-Cena D, Famoso-Perez P, Salom-Moreno J, et al. "Living an Obstacle Course": A qualitative study examining the experiences of caregivers of children with Rett Syndrome. *Int J Environ Res Public Health* 2018;16(1):25.
42. Mori Y, Downs J, Wong K, et al. Impacts of caring for a child with the CDKL5 disorder on parental wellbeing and family quality of life. *Orphanet J Rare Dis* 2017;12(1):16.
43. Lopez-Bastida J, Pena-Longobardo LM, Aranda-Reneo I, et al. Social/economic costs and health-related quality of life in patients with spinal muscular atrophy (SMA) in Spain. *Orphanet Journal of Rare Diseases* 2017;12 doi: 10.1186/s13023-017-0695-0
44. Flores D, Ribate MP, Montolio M, et al. Quantifying the economic impact of caregiving for Duchenne muscular dystrophy (DMD) in Spain. *European Journal of Health Economics* 2020;21(7):1015-23. doi: 10.1007/s10198-020-01197-6
45. Rice DB, Carboni-Jimenez A, Canedo-Ayala M, et al. Perceived Benefits and Faciliatators and Barriers to Providing Psychosocial Interventions for Informal Caregivers of People with Rare Diseases: A Scoping Review [Review]. *The Patient: Patient-Centered Outcomes Research* 2020;13(5):471-519.
46. Landfeldt E, Lindgren P, Bell CF, et al. Quantifying the burden of caregiving in Duchenne muscular dystrophy. *Journal of Neurology* 2016;263(5):906-15. doi: 10.1007/s00415-016-8080-9
47. Selman LE, Beynon T, Radcliffe E, et al. 'We're all carrying a burden that we're not sharing': a qualitative study of the impact of cutaneous T-cell lymphoma on the family. *British Journal of Dermatology* 2015;172(6):1581-92. doi: 10.1111/bjd.13583
48. Sloper T, Beresford B. Families with disabled children *BMJ* 2006;333(7575):928-29.
49. Hanbury A, Smith A.B., K B. Deriving Vignettes for the Rare Disease AADC Deficiency Using Parent, Caregiver and Clinician Interviews to Evaluate the Impact on Health-Related Quality of Life. *Patient Related Outcome Measures* 2021;12:1-12.
50. Currie G, Szabo J. "It is like a jungle gym, and everything is under construction": The parent's perspective of caring for a child with a rare disease. . *Child: Care, Health and Development* 2019;45(1):96-103.
51. Currie G, Szabo J. 'It would be much easier if we were just quiet and disappeared': Parents silenced in the experience of caring for children with rare diseases. *Health Expectations* 2019;22(6):1251-59.
52. Mutch K, Methley A, Hamid S, et al. If they are OK, we are OK: The experience of partners living with neuromyelitis optica. *Disability and Rehabilitation* 2017;39(13):1279-86.
53. McMullan J, Crowe A, Bailie C, et al. Improvements needed to support people living and working with a rare disease in Northern Ireland: current rare disease support perceived as inadequate. *Orphanet J Rare Dis* 2020;15(315)

For peer review only

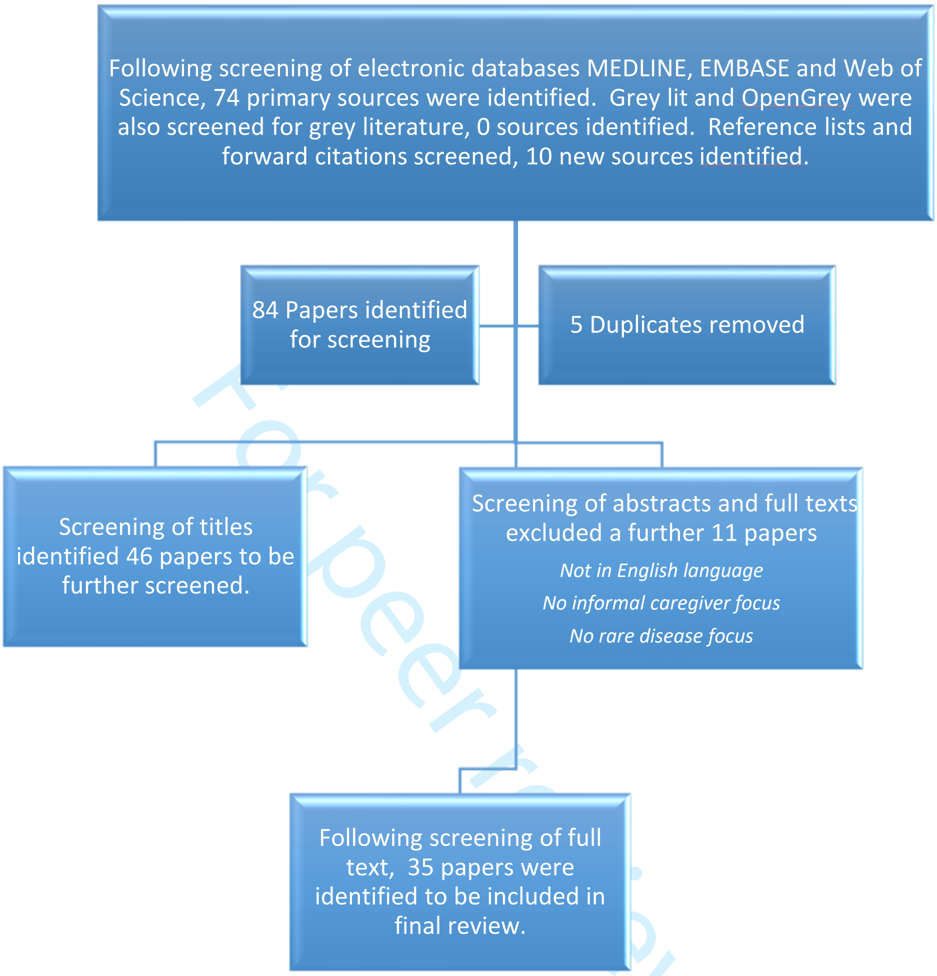

Figure 1 PRISMA flowchart

For peer review only

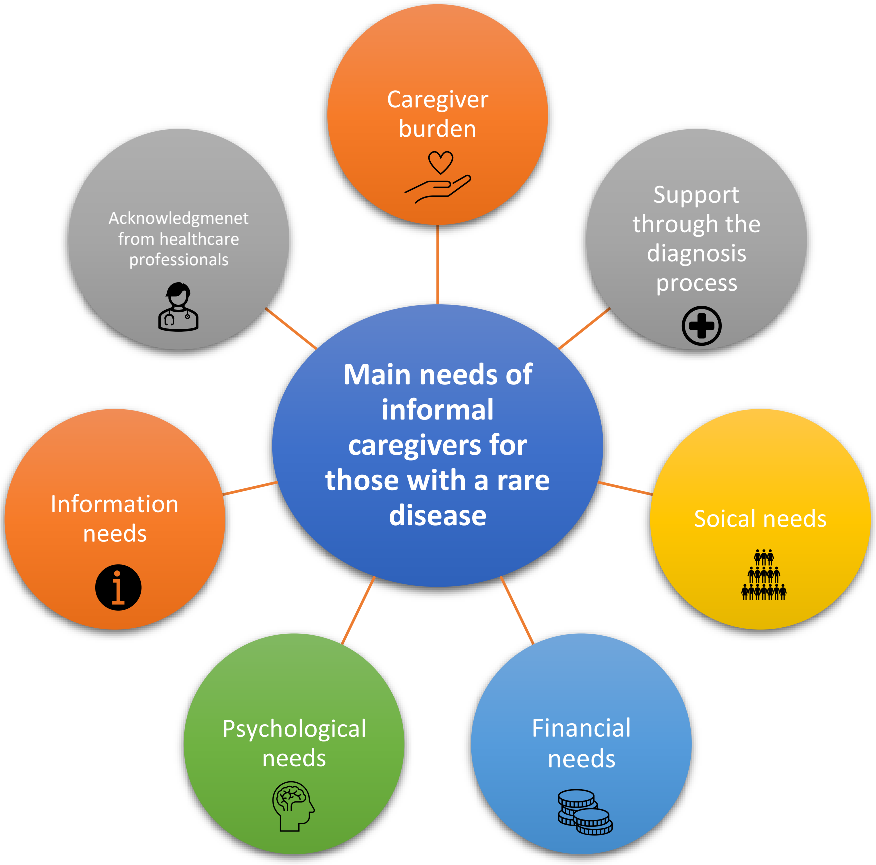

Figure 2 Main needs of caregivers for those with a rare disease

Supplementary file 1: Data extraction table				
**Author & year of publication**	**Country in which the study was conducted**	**Data collection method(s)**	**Participants**	**Identified caregiver needs**
Anderson, M., Elliott EJ, Zurynski YA.<br><br>2009	Australia	Survey	Parents/carers	• Support around diagnosis.<br>• Access to peer support groups.<br>• Psychological support.
Applebaum A.J., Polacek L.C., Walsh L. *et al.*<br><br>2020	USA	Survey and semi-structured interview	Caregivers of patients with Erdheim-Chester disease	• Social connections to prevent extreme isolation
Aubeeluck A.V. Buchanan H.E., Stupple E.J.N.<br><br>2011	England	Focus groups	Caregivers of those with Huntington's Disease	• More time to focus on themselves.
Craig T.J., Banjerji A., Riedl M.A., *et al.*<br><br>2021	USA	Online survey	Adults caring for an individual with Hereditary angioedema (HAE)	• Recognition for the important role they have.<br>• Inclusion in treatment discussions.
Hanbury A, Smith AB & Buesch K<br><br>2021	England	Vignettes were developed	Caregivers	• Engagement with research
Baumbusch, J., Mayer, S., & Sloan-Yip, I.<br><br>2018	Canada	Semi-structured interviews	Parents of children with a RD	• Acknowledgment from healthcare providers<br>• Support coordinating care<br>• Peer support – information and emotional<br>• Policies and programs to validate their role<br>• Support from genetic counsellor to connect them to others

For peer review only

Bendixen, R. M., & Houtrow, A.<br><br>2017	USA	Interviews	Parents of children with Duchenne muscular dystrophy	• Listened to by healthcare providers<br>• Inclusion in the diagnostic process<br>• Improved delivery of the diagnosis<br>• Improved follow up after diagnosis
Cañedo-Ayala, M., Rice, D. B., Levis, B., et al.<br><br>2020	USA	Questionnaire	Informal caregivers	• Mental health support
Currie, G., & Szabo, J.<br><br>2019	Canada	Semi-structured interviews	Parents of children with RDs	• Communication issues need addressed as parents often know more about the disease than healthcare providers.<br>• Improved coordination of care between providers and services caring for children with rare diseases.<br>• Gap in accessibility to government supports needs addressed.
Currie, G., & Szabo, J.<br><br>2019	Canada	Semi-structured interviews	Parents of children with rare neurodevelopmental diseases	• Health-care providers need more understanding of rare conditions.<br>• More support needed, parents often 'lose themselves' in this demanding caring role.
Flores D., Ribate M.P., Montolio M., *et al.*<br><br>2020	Spain	Questionnaires	Caregivers to patients with Duchenne muscular dystrophy	• Financial support.
Kanters T.A., van der Ploeg Ans. T, Brouwer W.B.F., *et al.*<br><br>2013	Netherlands	Questionnaire	Caregivers for those with Pompe disease.	• Support needed for informal caregivers.
Kasparian, N. A., Rutstein, A., Sansom-Daly, *et al.*	Australia	Telephone interviews	Patients and caregivers affected by Von Hippel-Lindau disease	• More supportive care services requested.

For peer review only

2015				
Landfelt E., Lindgren P., Bell C.F., *et al.*<br><br>2016	Germany, Italy, UK, USA	Visual analogue scale, survey, interview	Caregivers to patients with Duchenne muscular dystrophy	• Screening for depression.<br>• Holistic approach to family mental health needed.
Lagae, L., Irwin, J., Gibson, E., *et al.*<br><br>2019	Europe	Survey	Caregivers for those with Dravet syndrome (DS)	• Time needed to escape caring duties.<br>• Formal support and respite required.<br>• Stress is common – support needed.
Lopez-Bastida J., Pena-Longobardo L.M., Aranda-Reneo I, *et al.*<br><br>2017	Spain	Questionnaire	Patients and caregivers with Spinal Muscular Atrophy	• Financial support<br>• QoL must be addressed
Lyon, M. E., Thompkins, J. D., Fratantoni, K., *et al.*<br><br>2019	USA	Semi-structured interviews	Caregiving families	• Worries about the future need to be addressed.<br>• More time for themselves needed.<br>• Financial concerns must be addressed.
McKnight, A. J. M., Walker, R., Collins, C. (2020).	Northern Ireland	Report	N/A	• Access to accurate information<br>• Access to appropriate services<br>• Improved communication
McMullan, J., Crowe, A. L., Bailie, C. *et al.*<br><br>2020	Northern Ireland	Survey and semi-structured interviews	Rare disease collaborative groups	• Caregivers often overlooked in RD research – their opinions and experiences must be valued.
McMullan J, Crowe A.L., Downes K., *et al.*<br><br>2021	Northern Ireland	Survey and workshop	Caregivers of those with a rare disease	• Improved interactions with healthcare professionals.<br>• Improved emotional, psychological and social support.<br>• Assistance with finances.<br>• Better awareness of support services.
Mooney J., Graham K. & Watts R.A.	England	Semi-structured interviews	Patients with ANCA-associated vasculitis and their caregivers.	• Emotional support is needed.<br>• Reassurance about the future.

For peer review only

2019				
Mori, Y., Downs, J., Wong, K., *et al.*<br><br>2017	Australia	Survey	Families with a child with CDKL5 disorder	• Burden of daily caregiving requires support, particularly in relation to emotional wellbeing, sleep problems, financial difficulties, QoL.
Mutch, K., Methley, A., Hamid, S., *et al.*<br><br>2017	England	Semi-structured interviews	Partners of people with NMO	• Acknowledgement from HCP regarding the vital role they play in caring.
Palacios-Cena, D., Famoso-Perez, P., Salom-Moreno, J., *et al.*<br><br>2018	Spain	Interviews, focus groups, researcher's field notes, caregiver's personal documents	Caregivers of children with Rett Syndrome	• Answers needed regarding 'the first symptoms' and 'the need for a diagnosis'.<br>• Help with managing day to day life.<br>• Financial support.
Pelentsov, L. J., Fielder, A. L., & Esterman, A. J.<br><br>2016	Australia	Semi-structured focus group interviews	Parents of a child with a RD	• Social isolation must be addressed.<br>• Knowledge of HCP needs to be improved.<br>• Support needed as family relationship often impacted due to demands of caring.
Pelentsov, L. J., Laws, T. A., & Esterman, A. J.<br><br>2015	Australia	Scoping Review	Parents of child with a RD	• Improved parental supportive care – common unmet needs between RDs.
Rice D.B., Canedo-Ayala M., Carboni-Jimeniez A., Carrier M-E, *et al.*<br><br>2020	USA	Online questionnaire	Caregivers of people with SSc	• Emotional support is required.<br>• Help with physical needs.<br>• Interventions needed delivered through hardcopy or online resources, including those delivered after the care recipient's diagnosis.
Rice D.B., Carbino-Jimeniz A., Canedo-Ayala M. *et al.*<br><br>2020	USA	Scoping review	N/A	• Psychosocial interventions needed to reduce caregivers stress, burden and feelings of isolation among caregivers.

For peer review only

				• Future research should design interventions for caregivers.
Rodriguez A.A., Martinez O., Amayra I, *et al.*<br><br>2021	Spain	Questionnaire	Carers of children with neuromuscular disease	• Financial assistance.<br>• Employment support.<br>• Physical and psychological support.
Selman L.E., Beynon T., Radcliffe S., *et al.*<br><br>2014	London	Semi-structured qualitative interviews	Adult informal caregivers of patients with Cutaneous T-cell lymphoma	• Easily accessible services are needed that include the family in the unit of care, provide support and information, and understand the process of family adjustment and adaptation.
Sloper, T., & Beresford, B.<br><br>2006	UK	Report	Families of disabled children	• Policies and structures to support social and economic needs.
Somanadhan, S., & Larkin, P. J.<br><br>2016	Ireland	In-depth interviews	Parents of those with Mucopolysaccharidosis	• Reassurance and certainty needed about the future.
Wiblin, L., Durcan, R., Lee, M., *et al.*<br><br>2017	England	Qualitative in depth interviews	Patients and caregivers living with MSA and PSP	• Better connections to others to reduce social isolation.<br>• Improved communication.
Williams, J. K., Skirton, H., Paulsen, J. S., *et al.*<br><br>2009	USA, Canada	Focus groups	Adult caregivers of people with Huntington's disease	• Emotional distress support should be provided.<br>• Assistance in managing several roles.<br>• Mental health monitoring.
Wu, Y., Al-Janabi, H., Mallett, A., *et al.*<br><br>2020	Australia	Clinical data was used from Mitochondrial Disease, Epileptic	Parents of those with rare genetic conditions	• Health effects on family members need to be considered.

For peer review only

		Encephalopathy and Brain Malformation projects		

For peer review only

BMJ Open

The needs of informal caregivers of people with a rare disease: a rapid review of the literature.

Journal:	*BMJ Open*
Manuscript ID	bmjopen-2022-063263.R2
Article Type:	Original research
Date Submitted by the Author:	18-Oct-2022
Complete List of Authors:	Mcmullan, Julie; Queen's University Belfast Faculty of Medicine Health and Life Sciences, Centre for Public Health<br>Lohfeld, Lynne; Wenzhou Medical University Eye Hospital, ; Queen's University Belfast, Centre for Public Health<br>McKnight, Amy; Queen's University Belfast, Centre for Public Health
**Primary Subject Heading**:	Public health
Secondary Subject Heading:	Public health
Keywords:	Quality in health care < HEALTH SERVICES ADMINISTRATION & MANAGEMENT, Health policy < HEALTH SERVICES ADMINISTRATION & MANAGEMENT, PUBLIC HEALTH

SCHOLARONE™
Manuscripts

I, the Submitting Author has the right to grant and does grant on behalf of all authors of the Work (as defined in the below author licence), an exclusive licence and/or a non-exclusive licence for contributions from authors who are: i) UK Crown employees; ii) where BMJ has agreed a CC-BY licence shall apply, and/or iii) in accordance with the terms applicable for US Federal Government officers or employees acting as part of their official duties; on a worldwide, perpetual, irrevocable, royalty-free basis to BMJ Publishing Group Ltd ("BMJ") its licensees and where the relevant Journal is co-owned by BMJ to the co-owners of the Journal, to publish the Work in this journal and any other BMJ products and to exploit all rights, as set out in our licence.

The Submitting Author accepts and understands that any supply made under these terms is made by BMJ to the Submitting Author unless you are acting as an employee on behalf of your employer or a postgraduate student of an affiliated institution which is paying any applicable article publishing charge ("APC") for Open Access articles. Where the Submitting Author wishes to make the Work available on an Open Access basis (and intends to pay the relevant APC), the terms of reuse of such Open Access shall be governed by a Creative Commons licence – details of these licences and which Creative Commons licence will apply to this Work are set out in our licence referred to above.

Other than as permitted in any relevant BMJ Author's Self Archiving Policies, I confirm this Work has not been accepted for publication elsewhere, is not being considered for publication elsewhere and does not duplicate material already published. I confirm all authors consent to publication of this Work and authorise the granting of this licence.

For peer review only

1

The needs of informal caregivers of people with a rare disease: a rapid review of the literature.

Authors

Julie McMullan (PDRF) [1], Lynne Lohfeld (Lecturer) [1], Amy Jayne McKnight (Professor) [1]

Institutional address

[1] Centre for Public Health, School of Medicine Dentistry and Biomedical Sciences, Institute of Clinical Science Block A, Grosvenor Road, Belfast, BT12 6BA.

Corresponding author

Julie McMullan

Email: raredisease@qub.ac.uk

Abstract

Objectives: Many people living with a rare disease are cared for by a family member. Due to a frequent lack of individual rare disease (RD) knowledge from healthcare professionals, the patient and their informal caregiver are frequently obliged to become 'experts' in their specific condition. This puts huge strain on family life and results in caregivers juggling multiple roles in addition to unique caring roles including as advocate, case manager, and medical navigator. We conducted a rapid review of literature reporting on the unmet needs of Informal caregivers for people living with a RD. All searches were conducted on 14 September 2021 followed by a manual searches of reference lists on 21 September 2021.

Setting: Searches were conducted in Medline, Embase, Web of Science, GreyLit and OpenGrey.

Results: Thirty-five papers were included in the final review & data extracted. This rapid review presents several unmet needs identified by informal caregivers of persons with a RD. The related literature was organised thematically: caregiver burden, support through the diagnosis process, social needs, financial needs, psychological needs, information and communication needs and acknowledgement from healthcare professionals.

Conclusions: This review provides evidence that increased meaningful support is required for caregivers. Active engagement should be encouraged from this cohort in future research and

For peer review only

awareness raised of the support available to improving the quality of life for families living with an RD. The unmet needs identified through this review will benefit people living with a RD, caregivers, healthcare professionals and policy makers.

Key words: burden, informal caregiver, needs, rare disease, review.

Strengths and limitations

- This study explored a cohort of individuals who frequently go unnoticed and unreported in the literature – informal caregivers of individuals diagnosed with a rare disease.
- The rapid review followed the guidelines for conducting a rapid review of the literature.
- The review was conducted by experienced researchers in the area of RD carer needs who have a wealth of relevant experience. Additionally, manual searches of reference lists were conducted to identify any papers not found by the initial search strategy.
- It is important to acknowledge limitations of this review. Due to resource constraints, only one author (JM) initially screened the titles and abstracts from the total set of documents retrieved. This could lead to bias.
- Conducting a rapid review produced results quickly, including identifying the main self-identified needs of caregivers of people living with an RD. This is particularly important given the key role that informal caregivers play, and the fact that earlier research has focused on the person with an RD and not the caregiver.

The authors of this paper consider the needs of RD caregivers a priority and recognises the value they bring to the unique caring needs.

Introduction

In Europe, rare diseases (RDs) are those which affect less than 1 in 2000 people in a specified population [1]. Although each RD occurs infrequently, collectively RDs are a major public health issue affecting more than 450 million people globally [2 3]. RDs result in a wide variety of healthcare needs stemming from the involvement of multiple organ systems, such as respiratory complications, the circulatory system, muscular system, digestive system, and central nervous system. Many RDs are chronic, complex and associated with physical, intellectual, or neurological disabilities that significantly affect patients and their families. In addition, many families living with a RD lack peer and community support services [4-7]

For peer review only

Although not always the case, family often plays a pivotal role in a person's adjustment to chronic disease and is influenced by behavioural and social factors [8]. Caregivers are defined as individuals who provide care for a person in need/care recipient. On the other hand informal caregivers are defined as family members or close others who provide care for the person in need with no financial benefit in return. In essence, "formal caregivers" or "caregivers" is used to denote a category of professionals or semi-professionals who provide care with financial benefit. In contrast, "informal caregivers" are typically family members or friends who provide care but have no such benefits in return (in most European countries). Caring for someone with an RD affects many areas of life including psychologically, economically, physically and logistically [9]. The importance of good mental and physical health for the informal caregiver is therefore vital for their own wellbeing and to ensure they can sustain the essential role of assisting the person with an RD [10 11]. Individuals with RDs and their families often have limited evidence-based information to guide decisions about disease management and symptom relief [12 13]. Further, the inherent uncertainty that comes with having a RD, including delays in diagnosis (the average time for a diagnosis of a rare disease in Northern Ireland is 5 years) and a lack of knowledge about current and future care needs [14 15], impact access to services and management of the RD [16]. Research on the experience of having a RD indicates that care and service needs are often not based on the severity of the health condition. Rather, they are associated with poor quality of care and barriers to access leading to less satisfaction with healthcare services and care coordination [15 17].

Given the few people with a specific RD, healthcare providers often are not knowledgeable about the condition. Therefore people living with a RD and their carers have to become their own experts [18]. This causes a change to the usual patient-doctor relationship, which can bring challenges such as difficulties in communication and patients struggling to get sufficient accurate information to make informed choices [19 20].

Caring for someone with a RD can be highly demanding often requiring intense and unique care specific to the individuals needs [21]. Delayed diagnosis, lifelong caring, limited capacity for independent living, lack of treatment options and large health service needs have severe impacts on parent's physical and psychosocial wellbeing [22].

Rapid reviews are an emerging type of knowledge synthesis used to inform health-related policy decisions and discussions, especially when information needs are immediate [23-26]. Rapid reviews

For peer review only

4

streamline systematic review methods – for example, by focusing the literature search [23] while still aiming to produce valid conclusions. The requirements or the review, which was undertaken with a short deadline, were for a short but in-depth synthesis of the current state of the issues facing caregivers for those with a rare disease.

This review focuses on informal caregivers for people living with a RD. We were particularly interested in their holistic needs when caring for this unique population and how these may differ from other caring populations where there is often more support available e.g. carers of people with dementia or cancer.

Methodology

Search strategy and inclusion criteria

Three electronic databases were searched -- Medline, Embase and Web of Science -- using the combined terms ‘informal caregiver*’ and ‘rare disease*’. All searches were conducted on 14 September 2021, with no date restrictions. Reference lists of included papers were screened for further sources. A search was also conducted of grey literature using the databases GreyLit and OpenGrey. Duplicates and non-English language articles were excluded. The criteria for inclusion were articles that address caregiving for people living with an RD. Articles on caregiving alone or RD alone were excluded. A simplified list of databases and the number of papers retrieved is shown in Table 1.

Study selection and data extraction

Database searches were last conducted on 14 September 2021 by JM. Titles and abstracts of the identified articles were downloaded onto Endnote. Duplicate articles were removed and the remaining papers were screened through analysing their titles and abstracts. Additionally, manual searches of reference lists were conducted on 21 September 2021 to identify any papers not found by the initial search strategy. If relevant, the papers were then further screened by reading the full text. Data were extracted by JM who recorded the follow data for each study:

- Author & year of publication
- Country in which the study was conducted
- Data collection method(s)
- Participants
- Identified caregiver needs

For peer review only

5

The identified caregiver needs were extracted from the included papers [JM] and presented the data extraction table [AMcK, JM & LL] (Supplementary file 1). From this, common needs were grouped together under headings which became the themes for this review. The themes and content for the themes was agreed between the research team [AMcK, JM & LL].

Table 1: Literature search results

Database	No. of articles retrieved	No. of articles included in review
Medline	6	5
Embase	6	5
Web of Science	62	13
GreyLit	0	0
OpenGrey	0	0

This table shows the number of articles identified from each database before reference lists were checked.

An illustration of the search strategy including databases searched and screening methods is displayed in Figure 1, modelled on the PRISMA flow diagram.

Patient and Public Involvement

The need for this review was highlighted by a priorities workshop in 2020, which considered contributions from >2,000 individuals living with rare disease. The review was also shared with representatives from a local rare disease charity in Northern Ireland (the Northern Ireland Rare Disease Partnership).

Results

General description of the literature

Sources initially identified from each database were as follows: MEDLINE *n= 6*, Embase *n= 6*, Web of Science *n= 62*, OpenGrey *n=0* and GreyLit *n=0*. The screening process resulted in 30 documents which were reviewed based on their titles and abstracts. Following the screening 5 duplicate papers were removed and 25 papers were identified for full text screening. Further studies were identified from searching the reference lists. Finally, 35 texts were included in the full review, with characteristics of each source summarised from the completed data extraction table (Supplementary file 1).

For peer review only - http://bmjopen.bmj.com/site/about/guidelines.xhtml

Of the texts included, publication dates were from 2006 to 2021 and the studies were conducted in Australia, Canada, Germany, Ireland, Italy, Netherlands, Spain, United Kingdom and the United States. The identified studies focus on many rare diseases including Hereditary angioedema, Huntington's Disease, Duchenne muscular dystrophy and Von Hippel-Lindau disease.

A narrative review is presented below, highlighting the main needs of caregivers of people living with an RD. The related literature has been organised thematically under the following headings:

- Caregiver burden
- Support through the diagnosis process
- Social needs
- Financial needs
- Psychological needs
- Information and communication needs
- Acknowledgement from healthcare professionals

Findings from these studies were organised thematically under the following headings as shown visually in Figure 2.

Caregiver burden

Caring for someone with an RD presents a range of challenges [27 28] that are affected by the severity of the illness and its duration, knowledge about the condition and its changes over time, and one's ability to address the emotional toll involved with long-term care [29]. Despite the stress and difficulties associated with being a family caregiver [30], often they acknowledge the positive aspects of caring for their loved ones [30].

The quality of life of caregivers is often compromised by their placing others' needs before their own [31]. Previous research has shown that informal caregivers do not have enough time for themselves, [32] and that caring for someone with an RD can negatively impact on all dimensions of family life [33]. Informal caregivers are often forced to miss work or school days due to the demands of their role, frequently address unpleasant events and watch a loved one suffer [28]. Consequently, they would benefit greatly from support to reduce the burden of caring for example from support groups, respite

For peer review only

care and employment arrangements [34]. This includes both supports for informal carers of people with a variety of health issues, as well as for RD-related issues [34].

Support through the diagnosis process

In spite of marked medical progress over the past several decades in identifying an RD, there are substantial delays in diagnosing these conditions [35]. This can have adverse impacts on families, for example, financial and mental health [12]. Little attention is given to parent concern in the diagnostic process with previous research reporting concerns regarding how the diagnosis was delivered to parents, lack of guidance and poor follow-up post diagnosis [35]. Previous research has shown that families are often dissatisfied by the way in which a diagnosis is given, including an insensitive style of communication, not offering support or counselling, and inadequate provision of information about the disease. This can cause emotional stress for caregivers who have not yet learned about the condition and how it will affect them and their families. Therefore, interventions to support the following a diagnosis would be useful, delivered either in hardcopy or online [36].

Social needs

Social isolation is common among informal caregivers with the impact on personal relationships being a reoccurring theme [4 5]. All too often, informal caregivers have little time to themselves and lack appropriate support and time for respite. This in turn causes further emotional stress and in the case of parents, uncertainty about their child's future [37]. Parents of children with an RD often feel isolated from mainstream society and struggle to stay socially connected [4]. It was suggested that the insights gained through research studies regarding the impact on caregivers' social lives should be considered in future clinical service planning. Additionally holistic, empathic, and person-centred medical and psychosocial care is urgently needed for this cohort [6]. Support is needed as relationships among family members are often impacted due to demands of caring [4]. There is a need for improved parental supportive care as many common unmet needs exist across RDs [38].

Financial needs

Previous research has indicated that financial issues are a top concern of caregivers of persons with RD [30 6 39 27]. The high economic costs that families must cover means that financial burden is common among caregivers of those with an RD, many of whom are forced to exhaust their financial savings [40]. Purchasing equipment, hiring professionals, and the additional financial burden the illness has on families poses immense stress on family life [41]. Often financial issues impact on the ability of the caregiver to meet the individual's healthcare needs, increasing the strain on their lives [42]. Substantial

For peer review only - http://bmjopen.bmj.com/site/about/guidelines.xhtml

social/economic burden is mostly attributable to high direct non-healthcare costs [43]. Previous research has shown that caregivers' financial burden might be conditioned by the clinical condition of the patient [44]. In addition to the direct healthcare costs, caregivers of those with an RD report that their career choices are influenced due to their caring role. Furthermore, caring duties can result in missed working days due to emergency caring demands [37] making it difficult for many caregivers to maintain their job and advance in their careers [41]. Financial hardship adversely effects the mental health of caregivers [42].

Psychological needs

There is a lack of psychological support for families caring for children with a RD, and many report that accessing appropriate psychological care is difficult [6 12]. Previous research has suggested that this form of support should be offered at the time of diagnosis [12 10]. Depressive symptoms are often associated with caregiving burden and therefore there is a need to develop interventions in additional to promoting the existing validated tools for caregivers considering their special needs [10]. Caregivers often express concerns about the future [32], experiencing emotional distress that can compromise the well-being of family carers, who attempt to maintain multiple roles [11]. Psychological interventions can help reduce stress, a sense of being burdened and feelings of isolation that many RD caregivers feel [45]. Research has suggested that screening for depression is needed and emphasises the need for a holistic approach to family mental health in the context of chronic childhood disease [46]. Peer support is a key resource in terms of information and emotional support for parents who often begin their journey feeling isolated and alone [21].

Information and communication needs

Suggestions were made that information about the condition should be given to caregivers at the time of diagnosis as well as signposting them to groups connecting families who share common experiences [21]. Caregivers require access to accurate information, appropriate services and improved communication between patients, families, and a range of both health and social care and other public services [2]. Families require easily accessible services that include the family in the unit of care, provide support and information, and understand the process of family adjustment and adaptation in the long term [47]. Policies and structures are needed to support social and economic needs [48]. Engaging caregivers in future avenues of research is vital to ensure resources and funding are targeted in the best way [49].

Acknowledgement from healthcare professionals

For peer review only

9

Out of necessity, informal carers often know more about a particular RD than healthcare providers do. This often leads to poor communication and collaboration. Furthermore a lack of coordination of care force carers to fill the gap by juggling multiple roles including that of advocate, case manager, and medical navigator [50]. Caregivers often experience silencing or being silenced when interacting with health-care and social care systems and providers [51]. This has been attributed to the lack of knowledge about RDs by health-care providers who also are unaware of the impact that caring for someone with RD [50]. This points to the need to improve the knowledge of health care providers on the medical, social and financial impact these informal carers experience [4]. Health care providers should also acknowledge the vital role that informal caregivers play in promoting the health and quality of life of persons with an RD [52]. Caregivers, especially parents caring for someone with an RD, work hard to be heard and acquire services within health and social systems [50]. Another way that informal caregivers are silenced is due to how they are overlooked in the world of research despite growing emphasis on ensuring 'patient and public involvement'. It is vital that this pattern is changed so that they have an opportunity to be heard [53 27].

Discussion

The purpose of this rapid review was to synthesise and describe what is currently known about the needs of informal caregivers for people living with an RD. Based on the findings, several recommendations for future healthcare practices and policies, as well as for research are evident. First, it is important to consider the extreme burden often experienced by these caregivers, many of whom place the need of others ahead of their own while navigating this journey with little or no support [31]. Support is needed to help caregivers with many aspects of their caring duties from the initial diagnosis process which has been a difficult time for many families [35].

Social support would be welcomed to enable caregivers to have much needed time away from their caring duties to recharge and also to relieve them of the emotional strain which impacts on not only themselves but their wider family [4]. The mental strain of caregiving is evident from previous research, psychological interventions are required that consider the family as a whole to reduce the emotional strain and depression too often experienced by caregivers [21 45]. Many caregivers have also reported struggling financially not only due to the high costs of the practical side of caring but as a result of the impact caring can have on their ability to maintain a career [41], financial support therefore a priority.

Clear information should be provided for caregivers from diagnosis through to their rare disease journey [2] to replace the current situation of confusion and uncertainty due to unclear and incomplete

For peer review only - http://bmjopen.bmj.com/site/about/guidelines.xhtml

or conflicting messages. Lastly, caregivers must be recognised by healthcare professionals for the integral part they have in caring for someone with a rare disease. This relationship often changes the dynamic between health professional and patient and so caregivers must be listened to and viewed as an 'expert' [52]. All of the above-mentioned issues should be taken into consideration when planning future research, it is vital that informal caregivers views are valued.

Conclusion

This rapid review presents several unmet needs identified by informal caregivers of persons with a RD. It is hoped that the findings contribute to increasing the amount of meaningful support for caregivers as well as encourage their active participation in improving the quality of life for families living with an RD. It is important that this cohort are engaged in future research to ensure their needs are being addressed. The insights that are gained through future research in relation to the impact on caregivers' social lives should be considered in the priorities and strategic directions for clinical services. Interventions to support them following a diagnosis would be useful, delivered either in hardcopy or online. Better meeting needs of informal caregivers for people living with a RD is in the best interests of people with a RD, healthcare professionals and policy makers, as well as caregivers. It is important that awareness is raised about the range of support options that are available from health and social care providers, charities and/or support groups for informal caregivers of people with an RD [27].

Abbreviations

RD Rare disease

Declarations

- Ethics approval and consent to participate: Not applicable
- Consent for publication: Not applicable
- Availability of data and materials: Not applicable
- Competing interests: The authors declare that they have no competing interests.
- Funding: JM was supported by an award from the NI Public Health Agency and the Medical Research Council (MC_PC_16018) – Northern Ireland Executive support of the Northern Ireland Genomic Medicine Centre though Belfast Health and Social Care Trust.
- Authors' contributions: JM contributed to data curation, formal analysis, data interpretation, drafting and reviewing the original manuscript. LL & AJM contributed to study conception

For peer review only

and design, data interpretation, project administration, supervision and reviewing manuscript drafts. All authors approved the final version of the manuscript for submission.

- Acknowledgements: Not applicable

Reference list

1. Beaulieu CL, Majewski J, Schwartzentruber J, et al. FORGE Canada Consortium: outcomes of a 2-year national rare-disease gene discovery project. *American Journal of Human Genetics* 2014;94(6):809-17.
2. McKnight A, Walker R, Collins C. Communications and Information Review: NI Report for Rare Diseases, 2020.
3. McMullan J, Crowe A, Kerr K, et al. Perspectives on a NI Rare Disease Registry, 2020.
4. Pelentsov LJ, Fielder AL, Esterman AJ. The supportive care needs of parents with a child with a rare disease: A qualitative descriptive study. *Journal of Pediatric Nursing* 2016;31(3):e207-e18.
5. Wiblin L, Durcan R, Lee M, et al. The importance of connection to others in QoL in MSA and PSP. *Parkinsons Disease* 2017:1-9.
6. Kasparian NA, Rutstein A, Sansom-Daly UM, et al. Through the looking glass: An exploratory study of the lived experiences and unmet needs of families affected by Von Hippel-Lindau disease. *European Journal of Human Genetics* 2015;23(1):34-40.
7. Kole A, Faurisson F. The Voice of 12,000 Patients: Experiences and expectations of rare disease patients on diagnosis and care in Europe. Eurordis2009 [Available from: http://www.eurordis.org/publication/voice-12000-patients accessed 9 September 2021.
8. Buchanan H, Huang C. Health-related quality of life among informal caregivers assisting people with multiple sclerosis. *Disability and Rehabilitation* 2011;33(2):113-21.
9. Mulroy S, Robertson L, Aiberti K, et al. The impact of having a sibling with an intellectual disability: Parental perspectives in two disorders. *J Intellect Disabil Res* 2008;52:216-29.
10. Canedo-Ayala M, Rice DB, Levis B, et al. Factors associated with symptoms of depression among informal caregivers of people with systemic sclerosis: A cross-sectional study. *Disability and Rehabilitation* 2020;42(3):394-99.
11. Williams JK, Skirton H, Paulsen JS, et al. The emotional experiences of family carers in Huntington disease. *Journal of Advanced Nursing* 2009;65(4):789-98.
12. Anderson M, Elliott EJ, Zurynski YA. Australian families living with rare disease: experiences of diagnosis, health services use and needs for psychosocial support. *Orphanet J Rare Dis* 2013;8:22. doi: 10.1186/1750-1172-8-22 [published Online First: 2013/02/13]
13. Forsythe LP, Szydiowski V, Murad MH, et al. A systematic review of approaches for engaging patients for research on rare diseases. *Journal of General Internal Medicine* 2014;29(53):788-800.
14. Grut L, Kvam MH. Facing ignorance: people with rare disorders and their experiences with public health and welfare services. *Scandinavian Journal of Disability Research* 2013;15(1):20-32.
15. Huyard C. What if anything is specific about having a rare disorder? Patients' judgements on being ill and being rare. *Health Expectations* 2009;12(4)
16. Delive L, Samuelsson L, Tallborn A, et al. Stress and well-being among parents of children with rare diseases: a prospective intervention study. *Journal of Advanced Nursing* 2006;53(4):392-402.
17. Farmer JE, Marien WE, Clark MJ, et al. Primary care supports for children with chronic health conditions: identifying and predicting unmet family needs. *Journal of Pediatric Psychology* 2004;29(5):355-67.

For peer review only

18. Crowe A, McKnight A, McAneney H. Communication needs for individuals with rare diseases within and around the healthcare system of Northern Ireland. *Frontiers in Public Health* 2019;7(236)

19. Budych K, Helms TM, C. S. How do patients with rare diseases experience the medical encounter? Exploring the role behavior and its impact on patient-physician interaction. *Health Policy* 2012;105(2-3):154-64.

20. Dudding-Byth T. A powerful team: The family physican advocating for patients with a rare diease. *Australian Family Physician* 2015;44(9):634-38.

21. Baumbusch J, Mayer S, Sloan-Yip I. Alone in a crowd? Parents of children with rare diseases' experiences of navigating the healthcare system. *Journal of Genetic Counseling* 2018;28(1):80-90.

22. Madeo AC, K.E. OB, Bernhardt BA, et al. Factors associated with perceived uncertainty among parents of children with undiagnosed medical conditions. *American Journal of Medical Genetics* 2012;158A(8):1887-84.

23. Watt A, Cameron A, Sturm L, et al. Rapid reviews verus full systematic reviews: an inventory of current methods and practice in health technology assessment. . *International Journal of Technology Assessment in Health Care* 2008;24:133-39.

24. Ganann B, Ciliska D, Thomas H. Expediting systematic reviews: methods and implications of rapid reviews. . *Implementation Science* 2010;5(56)

25. Khangura S, Konnyu K, Cushman R, et al. Evidence summaries: the evolution of a rapid review approach. *Systematic reviews* 2012;1(10)

26. Konnya K, Kwok E, Skidmore B, et al. The effectiveness and safety of emergency department short stay units: a rapid review. *Open Medicine* 2012;6(e10-e16)

27. McMullan J, Crowe A, Downes K, et al. Carer reported experiences: Supporting someone with a rare disease. *Health and Social Care in the Community* 2021;0:1-12.

28. Craig TJ, Banerji A, Riedl MA, et al. Caregivers' role in managing hereditary angioedema and perceptions of treatment-related burden. *Allergy and Asthma Proceedings* 2021;42:S11-S16. doi: 10.2500/aap.2021.42.210029

29. Mooney J, Graham K, Watts RA. Impact of caring for someone with a rare rheumatic condition, views from patients and informal carers-the need for cat-like vigilance. *Rheumatology Advances in Practice* 2019;3(1) doi: 10.1093/rap/rkz003

30. Kanters TA, van der Ploeg AT, Brouwer WBF, et al. The impact of informal care for patients with Pompe disease: An application of the CarerQol instrument. *Molecular Genetics and Metabolism* 2013;110(3):281-86. doi: 10.1016/j.ymgme.2013.07.020

31. Aubeeluck AV, Buchanan H, Stupple EJN. 'All the burden on all the carers': Exploring quality of life with family caregivers of Huntington's disease patients. *Quality of Life Research* 2012;21(8):1425-35.

32. Lyon ME, Thompkins JD, Fratantoni K, et al. Family caregivers of children and adolescents with rare diseases: A novel palliative care intervention. *BMJ Supportive and Palliative Care* 2019;25(25)

33. Somanadhan S, Larkin PJ. Parents' experiences of living with, and caring for children, adolescents and young adults with Mucopolysaccharidosis (MPS). . *Orphanet J Rare Dis* 2016;11(1):138.

34. Wu Y, Sun F, Lee P. Family caregivers' lived experiences of caring for epidermolysis bullosa patients: A phenomenological study. *Journal of Clincial Nursing* 2020;29(9-10):1552-60.

35. Bendixen RM, Houtrow A. Parental reflections on the diagnostic process for duchenne muscular dystrophy: A qualitative study. *Journal of Pediatric Health Care* 2017;31(3):285-92.

36. Rice DB, Canedo-Ayala M, Carboni-Jimenez A, et al. Challenges and support service preferences of informal caregivers of people with systemic sclerosis: a cross-sectional survey. *Disability & Rehabiliation* 2020;42(16):2304-10.

37. Lagae L, Irwin J, Gibson E, et al. Caregiver impact and health service use in high and low severity Dravet syndrome: A multinational cohort study. *Seizure* 2019;65:72-77.

For peer review only - http://bmjopen.bmj.com/site/about/guidelines.xhtml

38. Pelentsov LJ, Laws TA, Esterman AJ. The supportive care needs of parents caring for a child with a rare disease: A scoping review. *Disability and Health Journal* 2015;8(4):475-91.
39. Rodriguez AA, Martinez O, Amayra I, et al. Diseases Costs and Impact of the Caring Role on Informal Carers of Children with Neuromuscular Disease. *International Journal of Environmental Research and Public Health* 2021;18(6) doi: 10.3390/ijerph18062991
40. Applebaum AJ, Polacek LC, Walsh L, et al. The unique burden of rare cancer caregiving: caregivers of patients with Erdheim-Chester disease. *Leukemia Lymphoma* 2020;61(6):1406-17.
41. Palacios-Cena D, Famoso-Perez P, Salom-Moreno J, et al. "Living an Obstacle Course": A qualitative study examining the experiences of caregivers of children with Rett Syndrome. *Int J Environ Res Public Health* 2018;16(1):25.
42. Mori Y, Downs J, Wong K, et al. Impacts of caring for a child with the CDKL5 disorder on parental wellbeing and family quality of life. *Orphanet J Rare Dis* 2017;12(1):16.
43. Lopez-Bastida J, Pena-Longobardo LM, Aranda-Reneo I, et al. Social/economic costs and health-related quality of life in patients with spinal muscular atrophy (SMA) in Spain. *Orphanet Journal of Rare Diseases* 2017;12 doi: 10.1186/s13023-017-0695-0
44. Flores D, Ribate MP, Montolio M, et al. Quantifying the economic impact of caregiving for Duchenne muscular dystrophy (DMD) in Spain. *European Journal of Health Economics* 2020;21(7):1015-23. doi: 10.1007/s10198-020-01197-6
45. Rice DB, Carboni-Jimenez A, Canedo-Ayala M, et al. Perceived Benefits and Faciliatators and Barriers to Providing Psychosocial Interventions for Informal Caregivers of People with Rare Diseases: A Scoping Review [Review]. *The Patient: Patient-Centered Outcomes Research* 2020;13(5):471-519.
46. Landfeldt E, Lindgren P, Bell CF, et al. Quantifying the burden of caregiving in Duchenne muscular dystrophy. *Journal of Neurology* 2016;263(5):906-15. doi: 10.1007/s00415-016-8080-9
47. Selman LE, Beynon T, Radcliffe E, et al. 'We're all carrying a burden that we're not sharing': a qualitative study of the impact of cutaneous T-cell lymphoma on the family. *British Journal of Dermatology* 2015;172(6):1581-92. doi: 10.1111/bjd.13583
48. Sloper T, Beresford B. Families with disabled children *BMJ* 2006;333(7575):928-29.
49. Hanbury A, Smith A.B., K B. Deriving Vignettes for the Rare Disease AADC Deficiency Using Parent, Caregiver and Clinician Interviews to Evaluate the Impact on Health-Related Quality of Life. *Patient Related Outcome Measures* 2021;12:1-12.
50. Currie G, Szabo J. “It is like a jungle gym, and everything is under construction”: The parent's perspective of caring for a child with a rare disease. . *Child: Care, Health and Development* 2019;45(1):96-103.
51. Currie G, Szabo J. 'It would be much easier if we were just quiet and disappeared': Parents silenced in the experience of caring for children with rare diseases. *Health Expectations* 2019;22(6):1251-59.
52. Mutch K, Methley A, Hamid S, et al. If they are OK, we are OK: The experience of partners living with neuromyelitis optica. *Disability and Rehabilitation* 2017;39(13):1279-86.
53. McMullan J, Crowe A, Bailie C, et al. Improvements needed to support people living and working with a rare disease in Northern Ireland: current rare disease support perceived as inadequate. *Orphanet J Rare Dis* 2020;15(315)

For peer review only

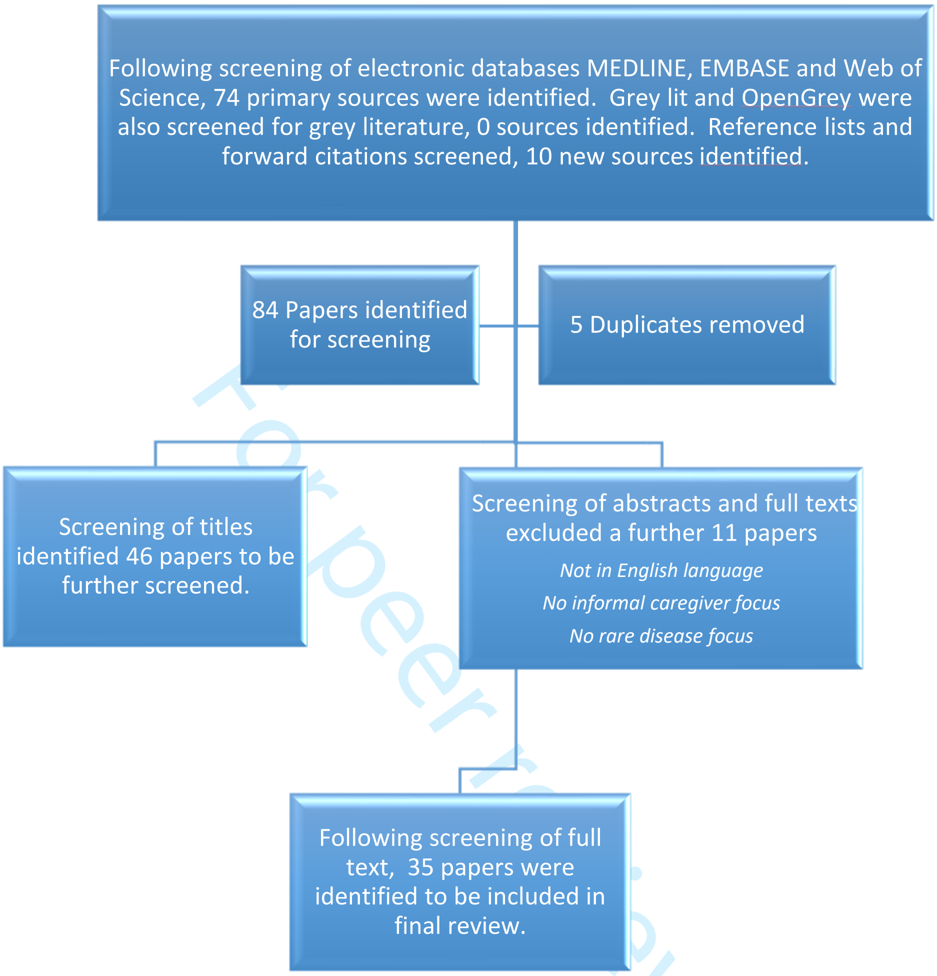

Figure 1 PRISMA flowchart

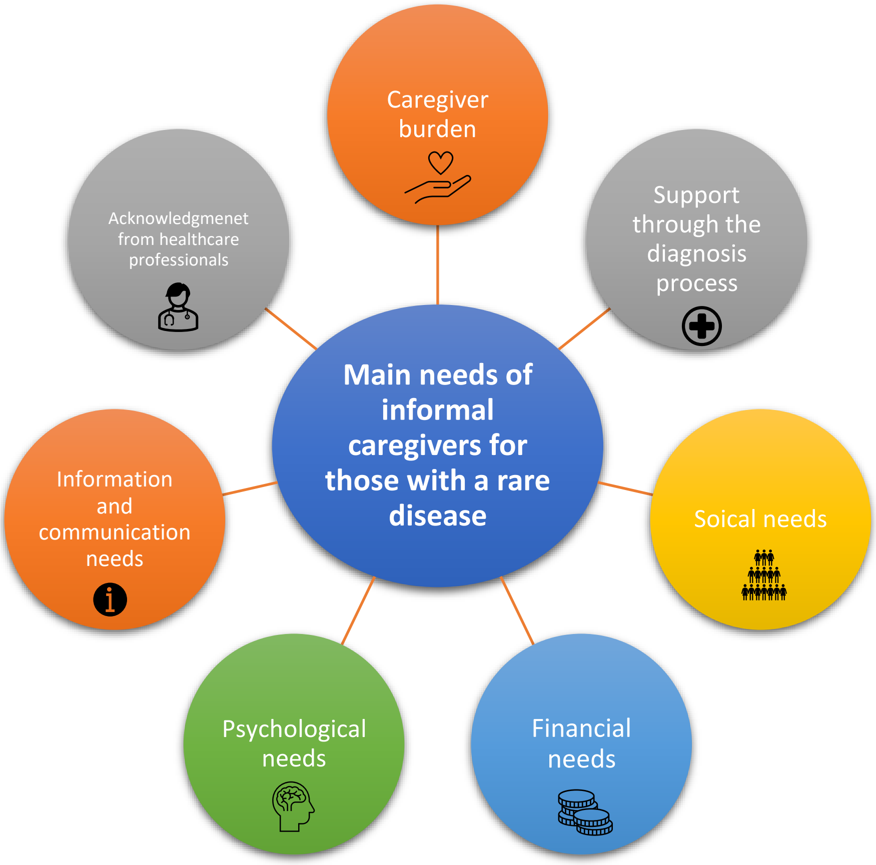

Figure 2 Main needs of caregivers for those with a rare disease

Supplementary file 1: Data extraction table				
**Author & year of publication**	**Country in which the study was conducted**	**Data collection method(s)**	**Participants**	**Identified caregiver needs**
Anderson, M., Elliott EJ, Zurynski YA.<br><br>2009	Australia	Survey	Parents/carers	• Support around diagnosis.<br>• Access to peer support groups.<br>• Psychological support.
Applebaum A.J., Polacek L.C., Walsh L. *et al.*<br><br>2020	USA	Survey and semi-structured interview	Caregivers of patients with Erdheim-Chester disease	• Social connections to prevent extreme isolation
Aubeeluck A.V. Buchanan H.E., Stupple E.J.N.<br><br>2011	England	Focus groups	Caregivers of those with Huntington's Disease	• More time to focus on themselves.
Craig T.J., Banjerji A., Riedl M.A., *et al.*<br><br>2021	USA	Online survey	Adults caring for an individual with Hereditary angioedema (HAE)	• Recognition for the important role they have.<br>• Inclusion in treatment discussions.
Hanbury A, Smith AB & Buesch K<br><br>2021	England	Vignettes were developed	Caregivers	• Engagement with research
Baumbusch, J., Mayer, S., & Sloan-Yip, I.<br><br>2018	Canada	Semi-structured interviews	Parents of children with a RD	• Acknowledgment from healthcare providers<br>• Support coordinating care<br>• Peer support – information and emotional<br>• Policies and programs to validate their role<br>• Support from genetic counsellor to connect them to others

For peer review only

Bendixen, R. M., & Houtrow, A.<br><br>2017	USA	Interviews	Parents of children with Duchenne muscular dystrophy	• Listened to by healthcare providers<br>• Inclusion in the diagnostic process<br>• Improved delivery of the diagnosis<br>• Improved follow up after diagnosis
Cañedo-Ayala, M., Rice, D. B., Levis, B., et al.<br><br>2020	USA	Questionnaire	Informal caregivers	• Mental health support
Currie, G., & Szabo, J.<br><br>2019	Canada	Semi-structured interviews	Parents of children with RDs	• Communication issues need addressed as parents often know more about the disease than healthcare providers.<br>• Improved coordination of care between providers and services caring for children with rare diseases.<br>• Gap in accessibility to government supports needs addressed.
Currie, G., & Szabo, J.<br><br>2019	Canada	Semi-structured interviews	Parents of children with rare neurodevelopmental diseases	• Health-care providers need more understanding of rare conditions.<br>• More support needed, parents often 'lose themselves' in this demanding caring role.
Flores D., Ribate M.P., Montolio M., *et al.*<br><br>2020	Spain	Questionnaires	Caregivers to patients with Duchenne muscular dystrophy	• Financial support.
Kanters T.A., van der Ploeg Ans. T, Brouwer W.B.F., *et al.*<br><br>2013	Netherlands	Questionnaire	Caregivers for those with Pompe disease.	• Support needed for informal caregivers.
Kasparian, N. A., Rutstein, A., Sansom-Daly, *et al.*	Australia	Telephone interviews	Patients and caregivers affected by Von Hippel-Lindau disease	• More supportive care services requested.

For peer review only

2015				
Landfelt E., Lindgren P., Bell C.F., *et al.*<br><br>2016	Germany, Italy, UK, USA	Visual analogue scale, survey, interview	Caregivers to patients with Duchenne muscular dystrophy	• Screening for depression.<br>• Holistic approach to family mental health needed.
Lagae, L., Irwin, J., Gibson, E., *et al.*<br><br>2019	Europe	Survey	Caregivers for those with Dravet syndrome (DS)	• Time needed to escape caring duties.<br>• Formal support and respite required.<br>• Stress is common – support needed.
Lopez-Bastida J., Pena-Longobardo L.M., Aranda-Reneo I, *et al.*<br><br>2017	Spain	Questionnaire	Patients and caregivers with Spinal Muscular Atrophy	• Financial support<br>• QoL must be addressed
Lyon, M. E., Thompkins, J. D., Fratantoni, K., *et al.*<br><br>2019	USA	Semi-structured interviews	Caregiving families	• Worries about the future need to be addressed.<br>• More time for themselves needed.<br>• Financial concerns must be addressed.
McKnight, A. J. M., Walker, R., Collins, C. (2020).	Northern Ireland	Report	N/A	• Access to accurate information<br>• Access to appropriate services<br>• Improved communication
McMullan, J., Crowe, A. L., Bailie, C. *et al.*<br><br>2020	Northern Ireland	Survey and semi-structured interviews	Rare disease collaborative groups	• Caregivers often overlooked in RD research – their opinions and experiences must be valued.
McMullan J, Crowe A.L., Downes K., *et al.*<br><br>2021	Northern Ireland	Survey and workshop	Caregivers of those with a rare disease	• Improved interactions with healthcare professionals.<br>• Improved emotional, psychological and social support.<br>• Assistance with finances.<br>• Better awareness of support services.
Mooney J., Graham K. & Watts R.A.	England	Semi-structured interviews	Patients with ANCA-associated vasculitis and their caregivers.	• Emotional support is needed.<br>• Reassurance about the future.

For peer review only

2019				
Mori, Y., Downs, J., Wong, K., *et al.* 2017	Australia	Survey	Families with a child with CDKL5 disorder	• Burden of daily caregiving requires support, particularly in relation to emotional wellbeing, sleep problems, financial difficulties, QoL.
Mutch, K., Methley, A., Hamid, S., *et al.* 2017	England	Semi-structured interviews	Partners of people with NMO	• Acknowledgement from HCP regarding the vital role they play in caring.
Palacios-Cena, D., Famoso-Perez, P., Salom-Moreno, J., *et al.* 2018	Spain	Interviews, focus groups, researcher's field notes, caregiver's personal documents	Caregivers of children with Rett Syndrome	• Answers needed regarding 'the first symptoms' and 'the need for a diagnosis'.<br>• Help with managing day to day life.<br>• Financial support.
Pelentsov, L. J., Fielder, A. L., & Esterman, A. J. 2016	Australia	Semi-structured focus group interviews	Parents of a child with a RD	• Social isolation must be addressed.<br>• Knowledge of HCP needs to be improved.<br>• Support needed as family relationship often impacted due to demands of caring.
Pelentsov, L. J., Laws, T. A., & Esterman, A. J. 2015	Australia	Scoping Review	Parents of child with a RD	• Improved parental supportive care – common unmet needs between RDs.
Rice D.B., Canedo-Ayala M., Carboni-Jimeniez A., Carrier M-E, *et al.* 2020	USA	Online questionnaire	Caregivers of people with SSc	• Emotional support is required.<br>• Help with physical needs.<br>• Interventions needed delivered through hardcopy or online resources, including those delivered after the care recipient's diagnosis.
Rice D.B., Carbino-Jimeniz A., Canedo-Ayala M. *et al.* 2020	USA	Scoping review	N/A	• Psychosocial interventions needed to reduce caregivers stress, burden and feelings of isolation among caregivers.

For peer review only

				• Future research should design interventions for caregivers.
Rodriguez A.A., Martinez O., Amayra I, *et al.*<br><br>2021	Spain	Questionnaire	Carers of children with neuromuscular disease	• Financial assistance.<br>• Employment support.<br>• Physical and psychological support.
Selman L.E., Beynon T., Radcliffe S., *et al.*<br><br>2014	London	Semi-structured qualitative interviews	Adult informal caregivers of patients with Cutaneous T-cell lymphoma	• Easily accessible services are needed that include the family in the unit of care, provide support and information, and understand the process of family adjustment and adaptation.
Sloper, T., & Beresford, B.<br><br>2006	UK	Report	Families of disabled children	• Policies and structures to support social and economic needs.
Somanadhan, S., & Larkin, P. J.<br><br>2016	Ireland	In-depth interviews	Parents of those with Mucopolysaccharidosis	• Reassurance and certainty needed about the future.
Wiblin, L., Durcan, R., Lee, M., *et al.*<br><br>2017	England	Qualitative in depth interviews	Patients and caregivers living with MSA and PSP	• Better connections to others to reduce social isolation.<br>• Improved communication.
Williams, J. K., Skirton, H., Paulsen, J. S., *et al.*<br><br>2009	USA, Canada	Focus groups	Adult caregivers of people with Huntington's disease	• Emotional distress support should be provided.<br>• Assistance in managing several roles.<br>• Mental health monitoring.
Wu, Y., Al-Janabi, H., Mallett, A., *et al.*<br><br>2020	Australia	Clinical data was used from Mitochondrial Disease, Epileptic	Parents of those with rare genetic conditions	• Health effects on family members need to be considered.

For peer review only

		Encephalopathy and Brain Malformation projects		

For peer review only

PRISMA 2020 Checklist

Section and Topic	Item #	Checklist item	Location where item is reported
**TITLE**			
Title	1	Identify the report as a systematic review.	N/A
**ABSTRACT**			
Abstract	2	See the PRISMA 2020 for Abstracts checklist.	N/A
**INTRODUCTION**			
Rationale	3	Describe the rationale for the review in the context of existing knowledge.	97-107
Objectives	4	Provide an explicit statement of the objective(s) or question(s) the review addresses.	104-107
**METHODS**			
Eligibility criteria	5	Specify the inclusion and exclusion criteria for the review and how studies were grouped for the syntheses.	111-118
Information sources	6	Specify all databases, registers, websites, organisations, reference lists and other sources searched or consulted to identify studies. Specify the date when each source was last searched or consulted.	111-126
Search strategy	7	Present the full search strategies for all databases, registers and websites, including any filters and limits used.	111-126
Selection process	8	Specify the methods used to decide whether a study met the inclusion criteria of the review, including how many reviewers screened each record and each report retrieved, whether they worked independently, and if applicable, details of automation tools used in the process.	121-137
Data collection process	9	Specify the methods used to collect data from reports, including how many reviewers collected data from each report, whether they worked independently, any processes for obtaining or confirming data from study investigators, and if applicable, details of automation tools used in the process.	122-139
Data items	10a	List and define all outcomes for which data were sought. Specify whether all results that were compatible with each outcome domain in each study were sought (e.g. for all measures, time points, analyses), and if not, the methods used to decide which results to collect.	N/A
	10b	List and define all other variables for which data were sought (e.g. participant and intervention characteristics, funding sources). Describe any assumptions made about any missing or unclear information.	N/A
Study risk of bias assessment	11	Specify the methods used to assess risk of bias in the included studies, including details of the tool(s) used, how many reviewers assessed each study and whether they worked independently, and if applicable, details of automation tools used in the process.	N/A
Effect measures	12	Specify for each outcome the effect measure(s) (e.g. risk ratio, mean difference) used in the synthesis or presentation of results.	N/A
Synthesis methods	13a	Describe the processes used to decide which studies were eligible for each synthesis (e.g. tabulating the study intervention characteristics and comparing against the planned groups for each synthesis (item #5)).	N/A
	13b	Describe any methods required to prepare the data for presentation or synthesis, such as handling of missing summary statistics, or data conversions.	N/A
	13c	Describe any methods used to tabulate or visually display results of individual studies and syntheses.	N/A
	13d	Describe any methods used to synthesize results and provide a rationale for the choice(s). If meta-analysis was performed, describe the model(s), method(s) to identify the presence and extent of statistical heterogeneity, and software package(s) used.	N/A
	13e	Describe any methods used to explore possible causes of heterogeneity among study results (e.g. subgroup analysis, meta-regression).	N/A
	13f	Describe any sensitivity analyses conducted to assess robustness of the synthesized results.	N/A
Reporting bias assessment	14	Describe any methods used to assess risk of bias due to missing results in a synthesis (arising from reporting biases).	N/A
Certainty	15	Describe any methods used to assess certainty (or confidence) in the body of evidence for an outcome.	N/A

For peer review only - http://bmjopen.bmj.com/site/about/guidelines.xhtml

PRISMA 2020 Checklist

Section and Topic	Item #	Checklist item	Location where item is reported
assessment			
**RESULTS**			
Study selection	16a	Describe the results of the search and selection process, from the number of records identified in the search to the number of studies included in the review, ideally using a flow diagram.	Figure 1
	16b	Cite studies that might appear to meet the inclusion criteria, but which were excluded, and explain why they were excluded.	114-117
Study characteristics	17	Cite each included study and present its characteristics.	Supp file 1
Risk of bias in studies	18	Present assessments of risk of bias for each included study.	N/A
Results of individual studies	19	For all outcomes, present, for each study: (a) summary statistics for each group (where appropriate) and (b) an effect estimate and its precision (e.g. confidence/credible interval), ideally using structured tables or plots.	N/A
Results of syntheses	20a	For each synthesis, briefly summarise the characteristics and risk of bias among contributing studies.	N/A
	20b	Present results of all statistical syntheses conducted. If meta-analysis was done, present for each the summary estimate and its precision (e.g. confidence/credible interval) and measures of statistical heterogeneity. If comparing groups, describe the direction of the effect.	N/A
	20c	Present results of all investigations of possible causes of heterogeneity among study results.	N/A
	20d	Present results of all sensitivity analyses conducted to assess the robustness of the synthesized results.	N/A
Reporting biases	21	Present assessments of risk of bias due to missing results (arising from reporting biases) for each synthesis assessed.	N/A
Certainty of evidence	22	Present assessments of certainty (or confidence) in the body of evidence for each outcome assessed.	N/A
**DISCUSSION**			
Discussion	23a	Provide a general interpretation of the results in the context of other evidence.	273-296
	23b	Discuss any limitations of the evidence included in the review.	38-51
	23c	Discuss any limitations of the review processes used.	38-51
	23d	Discuss implications of the results for practice, policy, and future research.	299-310
**OTHER INFORMATION**			
Registration and protocol	24a	Provide registration information for the review, including register name and registration number, or state that the review was not registered.	N/A
	24b	Indicate where the review protocol can be accessed, or state that a protocol was not prepared.	a review protocol was prepared and followed as described in the methods
	24c	Describe and explain any amendments to information provided at registration or in the protocol.	N/A
Support	25	Describe sources of financial or non-financial support for the review, and the role of the funders or sponsors in the review.	320-322
Competing	26	Declare any competing interests of review authors.	319

For peer review only - http://bmjopen.bmj.com/site/about/guidelines.xhtml

PRISMA 2020 Checklist

Section and Topic	Item #	Checklist item	Location where item is reported
interests			
Availability of data, code and other materials	27	Report which of the following are publicly available and where they can be found: template data collection forms; data extracted from included studies; data used for all analyses; analytic code; any other materials used in the review.	318

From: Page MJ, McKenzie JE, Bossuyt PM, Boutron I, Hoffmann TC, Mulrow CD, et al. The PRISMA 2020 statement: an updated guideline for reporting systematic reviews. BMJ 2021;372:n71. doi: 10.1136/bmj.n71

For more information, visit: http://www.prisma-statement.org/

For peer review only

BMJ Open

The needs of informal caregivers of people with a rare disease: a rapid review of the literature.

Journal:	*BMJ Open*
Manuscript ID	bmjopen-2022-063263.R3
Article Type:	Original research
Date Submitted by the Author:	08-Nov-2022
Complete List of Authors:	Mcmullan, Julie; Queen's University Belfast Faculty of Medicine Health and Life Sciences, Centre for Public Health<br>Lohfeld, Lynne; Wenzhou Medical University Eye Hospital, ; Queen's University Belfast, Centre for Public Health<br>McKnight, Amy; Queen's University Belfast, Centre for Public Health
**Primary Subject Heading**:	Public health
Secondary Subject Heading:	Public health
Keywords:	Quality in health care < HEALTH SERVICES ADMINISTRATION & MANAGEMENT, Health policy < HEALTH SERVICES ADMINISTRATION & MANAGEMENT, PUBLIC HEALTH

SCHOLARONE™
Manuscripts

I, the Submitting Author has the right to grant and does grant on behalf of all authors of the Work (as defined in the below author licence), an exclusive licence and/or a non-exclusive licence for contributions from authors who are: i) UK Crown employees; ii) where BMJ has agreed a CC-BY licence shall apply, and/or iii) in accordance with the terms applicable for US Federal Government officers or employees acting as part of their official duties; on a worldwide, perpetual, irrevocable, royalty-free basis to BMJ Publishing Group Ltd ("BMJ") its licensees and where the relevant Journal is co-owned by BMJ to the co-owners of the Journal, to publish the Work in this journal and any other BMJ products and to exploit all rights, as set out in our licence.

The Submitting Author accepts and understands that any supply made under these terms is made by BMJ to the Submitting Author unless you are acting as an employee on behalf of your employer or a postgraduate student of an affiliated institution which is paying any applicable article publishing charge ("APC") for Open Access articles. Where the Submitting Author wishes to make the Work available on an Open Access basis (and intends to pay the relevant APC), the terms of reuse of such Open Access shall be governed by a Creative Commons licence – details of these licences and which Creative Commons licence will apply to this Work are set out in our licence referred to above.

Other than as permitted in any relevant BMJ Author's Self Archiving Policies, I confirm this Work has not been accepted for publication elsewhere, is not being considered for publication elsewhere and does not duplicate material already published. I confirm all authors consent to publication of this Work and authorise the granting of this licence.

For peer review only

The needs of informal caregivers of people with a rare disease: a rapid review of the literature.

Authors

Julie McMullan (PDRF) [1], Lynne Lohfeld (Lecturer) [1], Amy Jayne McKnight (Professor) [1]

Institutional address

[1] Centre for Public Health, School of Medicine Dentistry and Biomedical Sciences, Institute of Clinical Science Block A, Grosvenor Road, Belfast, BT12 6BA.

Corresponding author

Julie McMullan

Email: raredisease@qub.ac.uk

For peer review only

Abstract

Objectives: Many people living with a rare disease are cared for by a family member. Due to a frequent lack of individual rare disease (RD) knowledge from healthcare professionals, the patient and their informal caregiver are frequently obliged to become 'experts' in their specific condition. This puts huge strain on family life and results in caregivers juggling multiple roles in addition to unique caring roles including as advocate, case manager, and medical navigator. We conducted a rapid review of literature reporting on the unmet needs of Informal caregivers for people living with a RD. All searches were conducted on 14 September 2021 followed by a manual searches of reference lists on 21 September 2021.

Setting: Searches were conducted in Medline, Embase, Web of Science, GreyLit and OpenGrey.

Results: Thirty-five papers were included in the final review & data extracted. This rapid review presents several unmet needs identified by informal caregivers of persons with a RD. The related literature was organised thematically: caregiver burden, support through the diagnosis process, social needs, financial needs, psychological needs, information and communication needs and acknowledgement from healthcare professionals.

Conclusions: This review provides evidence that increased meaningful support is required for caregivers. Active engagement should be encouraged from this cohort in future research and

For peer review only - http://bmjopen.bmj.com/site/about/guidelines.xhtml

awareness raised of the support available to improving the quality of life for families living with an RD. The unmet needs identified through this review will benefit people living with a RD, caregivers, healthcare professionals and policy makers.

Key words: burden, informal caregiver, needs, rare disease, review.

Strengths and limitations

- This study explored a cohort of individuals who frequently go unnoticed and unreported in the literature – informal caregivers of individuals diagnosed with a rare disease.
- The rapid review followed the guidelines for conducting a rapid review of the literature.
- The review was conducted by experienced researchers in the area of RD carer needs who have a wealth of relevant experience. Additionally, manual searches of reference lists were conducted to identify any papers not found by the initial search strategy.
- It is important to acknowledge limitations of this review. Due to resource constraints, only one author (JM) initially screened the titles and abstracts from the total set of documents retrieved. This could lead to bias.
- Conducting a rapid review produced results quickly, including identifying the main self-identified needs of caregivers of people living with an RD. This is particularly important given the key role that informal caregivers play, and the fact that earlier research has focused on the person with an RD and not the caregiver.

The authors of this paper consider the needs of RD caregivers a priority and recognises the value they bring to the unique caring needs.

Introduction

In Europe, rare diseases (RDs) are those which affect less than 1 in 2000 people in a specified population [1]. Although each RD occurs infrequently, collectively RDs are a major public health issue affecting more than 450 million people globally [2 3]. RDs result in a wide variety of healthcare needs stemming from the involvement of multiple organ systems, such as respiratory complications, the circulatory system, muscular system, digestive system, and central nervous system. Many RDs are chronic, complex and associated with physical, intellectual, or neurological disabilities that significantly affect patients and their families. In addition, many families living with a RD lack peer and community support services [4-7]

For peer review only

Although not always the case, family often plays a pivotal role in a person's adjustment to chronic disease and is influenced by behavioural and social factors [8]. Caregivers are defined as individuals who provide care for a person in need/care recipient. On the other hand informal caregivers are defined as family members or close others who provide care for the person in need with no financial benefit in return. In essence, "formal caregivers" or "caregivers" is used to denote a category of professionals or semi-professionals who provide care with financial benefit. In contrast, "informal caregivers" are typically family members or friends who provide care but have no such benefits in return (in most European countries). Caring for someone with an RD affects many areas of life including psychologically, economically, physically and logistically [9]. The importance of good mental and physical health for the informal caregiver is therefore vital for their own wellbeing and to ensure they can sustain the essential role of assisting the person with an RD [10 11]. Individuals with RDs and their families often have limited evidence-based information to guide decisions about disease management and symptom relief [12 13]. Further, the inherent uncertainty that comes with having a RD, including delays in diagnosis (the average time for a diagnosis of a rare disease in Northern Ireland is 5 years) and a lack of knowledge about current and future care needs [14 15], impact access to services and management of the RD [16]. Research on the experience of having a RD indicates that care and service needs are often not based on the severity of the health condition. Rather, they are associated with poor quality of care and barriers to access leading to less satisfaction with healthcare services and care coordination [15 17].

Given the few people with a specific RD, healthcare providers often are not knowledgeable about the condition. Therefore people living with a RD and their carers have to become their own experts [18]. This causes a change to the usual patient-doctor relationship, which can bring challenges such as difficulties in communication and patients struggling to get sufficient accurate information to make informed choices [19 20].

Caring for someone with a RD can be highly demanding often requiring intense and unique care specific to the individuals needs [21]. Delayed diagnosis, lifelong caring, limited capacity for independent living, lack of treatment options and large health service needs have severe impacts on parent's physical and psychosocial wellbeing [22].

Rapid reviews are an emerging type of knowledge synthesis used to inform health-related policy decisions and discussions, especially when information needs are immediate [23-26]. Rapid reviews

For peer review only

streamline systematic review methods – for example, by focusing the literature search [23] while still aiming to produce valid conclusions. The requirements or the review, which was undertaken with a short deadline, were for a short but in-depth synthesis of the current state of the issues facing caregivers for those with a rare disease.

This review focuses on informal caregivers for people living with a RD. We were particularly interested in their holistic needs when caring for this unique population and how these may differ from other caring populations where there is often more support available e.g. carers of people with dementia or cancer.

Methodology

Search strategy and inclusion criteria

Three electronic databases were searched -- Medline, Embase and Web of Science -- using the combined terms 'informal caregiver*' and 'rare disease*' (Supplementary file 1). All searches were conducted on 14 September 2021, with no date restrictions. Reference lists of included papers were screened for further sources. A search was also conducted of grey literature using the databases GreyLit and OpenGrey. Duplicates and non-English language articles were excluded. The criteria for inclusion were articles that address caregiving for people living with an RD. Articles on caregiving alone or RD alone were excluded. A simplified list of databases and the number of papers retrieved is shown in Table 1.

Study selection and data extraction

Database searches were last conducted on 14 September 2021 by JM. Titles and abstracts of the identified articles were downloaded onto Endnote. Duplicate articles were removed and the remaining papers were screened through analysing their titles and abstracts. Additionally, manual searches of reference lists were conducted on 21 September 2021 to identify any papers not found by the initial search strategy. If relevant, the papers were then further screened by reading the full text. Data were extracted by JM who recorded the follow data for each study:

- Author & year of publication
- Country in which the study was conducted
- Data collection method(s)
- Participants
- Identified caregiver needs

For peer review only

5

The identified caregiver needs were extracted from the included papers [JM] and presented the data extraction table [AMcK, JM & LL] (Supplementary file 2). From this, common needs were grouped together under headings which became the themes for this review. The themes and content for the themes was agreed between the research team [AMcK, JM & LL].

Table 1: Literature search results

Database	No. of articles retrieved	No. of articles included in review
Medline	6	5
Embase	6	5
Web of Science	62	13
GreyLit	0	0
OpenGrey	0	0

This table shows the number of articles identified from each database before reference lists were checked.

An illustration of the search strategy including databases searched and screening methods is displayed in Figure 1, modelled on the PRISMA flow diagram.

Patient and Public Involvement

The need for this review was highlighted by a priorities workshop in 2020, which considered contributions from >2,000 individuals living with rare disease. The review was also shared with representatives from a local rare disease charity in Northern Ireland (the Northern Ireland Rare Disease Partnership).

Results

General description of the literature

Sources initially identified from each database were as follows: MEDLINE *n= 6*, Embase *n= 6*, Web of Science *n= 62*, OpenGrey *n=0* and GreyLit *n=0*. The screening process resulted in 30 documents which were reviewed based on their titles and abstracts. Following the screening 5 duplicate papers were removed and 25 papers were identified for full text screening. Further studies were identified from searching the reference lists. Finally, 35 texts were included in the full review, with characteristics of each source summarised from the completed data extraction table (Supplementary file 2).

Of the texts included, publication dates were from 2006 to 2021 and the studies were conducted in Australia, Canada, Germany, Ireland, Italy, Netherlands, Spain, United Kingdom and the United States. The identified studies focus on many rare diseases including Hereditary angioedema, Huntington's Disease, Duchenne muscular dystrophy and Von Hippel-Lindau disease.

A narrative review is presented below, highlighting the main needs of caregivers of people living with an RD. The related literature has been organised thematically under the following headings:

- Caregiver burden
- Support through the diagnosis process
- Social needs
- Financial needs
- Psychological needs
- Information and communication needs
- Acknowledgement from healthcare professionals

Findings from these studies were organised thematically under the following headings as shown visually in Figure 2.

Caregiver burden

Caring for someone with an RD presents a range of challenges [27 28] that are affected by the severity of the illness and its duration, knowledge about the condition and its changes over time, and one's ability to address the emotional toll involved with long-term care [29]. Despite the stress and difficulties associated with being a family caregiver [30], often they acknowledge the positive aspects of caring for their loved ones [30].

The quality of life of caregivers is often compromised by their placing others' needs before their own [31]. Previous research has shown that informal caregivers do not have enough time for themselves, [32] and that caring for someone with an RD can negatively impact on all dimensions of family life [33]. Informal caregivers are often forced to miss work or school days due to the demands of their role, frequently address unpleasant events and watch a loved one suffer [28]. Consequently, they would benefit greatly from support to reduce the burden of caring for example from support groups, respite

For peer review only

care and employment arrangements [34]. This includes both supports for informal carers of people with a variety of health issues, as well as for RD-related issues [34].

Support through the diagnosis process

In spite of marked medical progress over the past several decades in identifying an RD, there are substantial delays in diagnosing these conditions [35]. This can have adverse impacts on families, for example, financial and mental health [12]. Little attention is given to parent concern in the diagnostic process with previous research reporting concerns regarding how the diagnosis was delivered to parents, lack of guidance and poor follow-up post diagnosis [35]. Previous research has shown that families are often dissatisfied by the way in which a diagnosis is given, including an insensitive style of communication, not offering support or counselling, and inadequate provision of information about the disease. This can cause emotional stress for caregivers who have not yet learned about the condition and how it will affect them and their families. Therefore, interventions to support the following a diagnosis would be useful, delivered either in hardcopy or online [36].

Social needs

Social isolation is common among informal caregivers with the impact on personal relationships being a reoccurring theme [4 5]. All too often, informal caregivers have little time to themselves and lack appropriate support and time for respite. This in turn causes further emotional stress and in the case of parents, uncertainty about their child's future [37]. Parents of children with an RD often feel isolated from mainstream society and struggle to stay socially connected [4]. It was suggested that the insights gained through research studies regarding the impact on caregivers' social lives should be considered in future clinical service planning. Additionally holistic, empathic, and person-centred medical and psychosocial care is urgently needed for this cohort [6]. Support is needed as relationships among family members are often impacted due to demands of caring [4]. There is a need for improved parental supportive care as many common unmet needs exist across RDs [38].

Financial needs

Previous research has indicated that financial issues are a top concern of caregivers of persons with RD [30 6 39 27]. The high economic costs that families must cover means that financial burden is common among caregivers of those with an RD, many of whom are forced to exhaust their financial savings [40]. Purchasing equipment, hiring professionals, and the additional financial burden the illness has on families poses immense stress on family life [41]. Often financial issues impact on the ability of the caregiver to meet the individual's healthcare needs, increasing the strain on their lives [42]. Substantial

For peer review only - http://bmjopen.bmj.com/site/about/guidelines.xhtml

social/economic burden is mostly attributable to high direct non-healthcare costs [43]. Previous research has shown that caregivers' financial burden might be conditioned by the clinical condition of the patient [44]. In addition to the direct healthcare costs, caregivers of those with an RD report that their career choices are influenced due to their caring role. Furthermore, caring duties can result in missed working days due to emergency caring demands [37] making it difficult for many caregivers to maintain their job and advance in their careers [41]. Financial hardship adversely effects the mental health of caregivers [42].

Psychological needs

There is a lack of psychological support for families caring for children with a RD, and many report that accessing appropriate psychological care is difficult [6 12]. Previous research has suggested that this form of support should be offered at the time of diagnosis [12 10]. Depressive symptoms are often associated with caregiving burden and therefore there is a need to develop interventions in additional to promoting the existing validated tools for caregivers considering their special needs [10]. Caregivers often express concerns about the future [32], experiencing emotional distress that can compromise the well-being of family carers, who attempt to maintain multiple roles [11]. Psychological interventions can help reduce stress, a sense of being burdened and feelings of isolation that many RD caregivers feel [45]. Research has suggested that screening for depression is needed and emphasises the need for a holistic approach to family mental health in the context of chronic childhood disease [46]. Peer support is a key resource in terms of information and emotional support for parents who often begin their journey feeling isolated and alone [21].

Information and communication needs

Suggestions were made that information about the condition should be given to caregivers at the time of diagnosis as well as signposting them to groups connecting families who share common experiences [21]. Caregivers require access to accurate information, appropriate services and improved communication between patients, families, and a range of both health and social care and other public services [2]. Families require easily accessible services that include the family in the unit of care, provide support and information, and understand the process of family adjustment and adaptation in the long term [47]. Policies and structures are needed to support social and economic needs [48]. Engaging caregivers in future avenues of research is vital to ensure resources and funding are targeted in the best way [49].

Acknowledgement from healthcare professionals

For peer review only

9

Out of necessity, informal carers often know more about a particular RD than healthcare providers do. This often leads to poor communication and collaboration. Furthermore a lack of coordination of care force carers to fill the gap by juggling multiple roles including that of advocate, case manager, and medical navigator [50]. Caregivers often experience silencing or being silenced when interacting with health-care and social care systems and providers [51]. This has been attributed to the lack of knowledge about RDs by health-care providers who also are unaware of the impact that caring for someone with RD [50]. This points to the need to improve the knowledge of health care providers on the medical, social and financial impact these informal carers experience [4]. Health care providers should also acknowledge the vital role that informal caregivers play in promoting the health and quality of life of persons with an RD [52]. Caregivers, especially parents caring for someone with an RD, work hard to be heard and acquire services within health and social systems [50]. Another way that informal caregivers are silenced is due to how they are overlooked in the world of research despite growing emphasis on ensuring 'patient and public involvement'. It is vital that this pattern is changed so that they have an opportunity to be heard [53 27].

Discussion

The purpose of this rapid review was to synthesise and describe what is currently known about the needs of informal caregivers for people living with an RD. Based on the findings, several recommendations for future healthcare practices and policies, as well as for research are evident. First, it is important to consider the extreme burden often experienced by these caregivers, many of whom place the need of others ahead of their own while navigating this journey with little or no support [31]. Support is needed to help caregivers with many aspects of their caring duties from the initial diagnosis process which has been a difficult time for many families [35].

Social support would be welcomed to enable caregivers to have much needed time away from their caring duties to recharge and also to relieve them of the emotional strain which impacts on not only themselves but their wider family [4]. The mental strain of caregiving is evident from previous research, psychological interventions are required that consider the family as a whole to reduce the emotional strain and depression too often experienced by caregivers [21 45]. Many caregivers have also reported struggling financially not only due to the high costs of the practical side of caring but as a result of the impact caring can have on their ability to maintain a career [41], financial support therefore a priority.

Clear information should be provided for caregivers from diagnosis through to their rare disease journey [2] to replace the current situation of confusion and uncertainty due to unclear and incomplete

For peer review only

or conflicting messages. Lastly, caregivers must be recognised by healthcare professionals for the integral part they have in caring for someone with a rare disease. This relationship often changes the dynamic between health professional and patient and so caregivers must be listened to and viewed as an 'expert' [52]. All of the above-mentioned issues should be taken into consideration when planning future research, it is vital that informal caregivers views are valued.

Conclusion

This rapid review presents several unmet needs identified by informal caregivers of persons with a RD. It is hoped that the findings contribute to increasing the amount of meaningful support for caregivers as well as encourage their active participation in improving the quality of life for families living with an RD. It is important that this cohort are engaged in future research to ensure their needs are being addressed. The insights that are gained through future research in relation to the impact on caregivers' social lives should be considered in the priorities and strategic directions for clinical services. Interventions to support them following a diagnosis would be useful, delivered either in hardcopy or online. Better meeting needs of informal caregivers for people living with a RD is in the best interests of people with a RD, healthcare professionals and policy makers, as well as caregivers. It is important that awareness is raised about the range of support options that are available from health and social care providers, charities and/or support groups for informal caregivers of people with an RD [27].

Figure legends

Figure 1 PRISMA flowchart

Figure 2 Main needs of caregivers for those with a rare disease

Abbreviations

RD Rare disease

Declarations

- Ethics approval and consent to participate: This study does not involve human participants and ethical approval was not required
- Consent for publication: Not applicable
- Availability of data and materials: Not applicable
- Competing interests: The authors declare that they have no competing interests.

For peer review only

- Funding: JM was supported by an award from the NI Public Health Agency and the Medical Research Council (MC_PC_16018) – Northern Ireland Executive support of the Northern Ireland Genomic Medicine Centre though Belfast Health and Social Care Trust.
- Authors' contributions: JM contributed to data curation, formal analysis, data interpretation, drafting and reviewing the original manuscript. LL & AJM contributed to study conception and design, data interpretation, project administration, supervision and reviewing manuscript drafts. All authors approved the final version of the manuscript for submission.
- Acknowledgements: Not applicable

Reference list

1. Beaulieu CL, Majewski J, Schwartzentruber J, et al. FORGE Canada Consortium: outcomes of a 2-year national rare-disease gene discovery project. *American Journal of Human Genetics* 2014;94(6):809-17.
2. McKnight A, Walker R, Collins C. Communications and Information Review: NI Report for Rare Diseases, 2020.
3. McMullan J, Crowe A, Kerr K, et al. Perspectives on a NI Rare Disease Registry, 2020.
4. Pelentsov LJ, Fielder AL, Esterman AJ. The supportive care needs of parents with a child with a rare disease: A qualitative descriptive study. *Journal of Pediatric Nursing* 2016;31(3):e207-e18.
5. Wiblin L, Durcan R, Lee M, et al. The importance of connection to others in QoL in MSA and PSP. *Parkinsons Disease* 2017:1-9.
6. Kasparian NA, Rutstein A, Sansom-Daly UM, et al. Through the looking glass: An exploratory study of the lived experiences and unmet needs of families affected by Von Hippel-Lindau disease. *European Journal of Human Genetics* 2015;23(1):34-40.
7. Kole A, Faurisson F. The Voice of 12,000 Patients: Experiences and expectations of rare disease patients on diagnosis and care in Europe. Eurordis2009 [Available from: http://www.eurordis.org/publication/voice-12000-patients accessed 9 September 2021.
8. Buchanan H, Huang C. Health-related quality of life among informal caregivers assisting people with multiple sclerosis. *Disability and Rehabilitation* 2011;33(2):113-21.
9. Mulroy S, Robertson L, Aiberti K, et al. The impact of having a sibling with an intellectual disability: Parental perspectives in two disorders. *J Intellect Disabil Res* 2008;52:216-29.
10. Canedo-Ayala M, Rice DB, Levis B, et al. Factors associated with symptoms of depression among informal caregivers of people with systemic sclerosis: A cross-sectional study. *Disability and Rehabilitation* 2020;42(3):394-99.
11. Williams JK, Skirton H, Paulsen JS, et al. The emotional experiences of family carers in Huntington disease. *Journal of Advanced Nursing* 2009;65(4):789-98.
12. Anderson M, Elliott EJ, Zurynski YA. Australian families living with rare disease: experiences of diagnosis, health services use and needs for psychosocial support. *Orphanet J Rare Dis* 2013;8:22. doi: 10.1186/1750-1172-8-22 [published Online First: 2013/02/13]
13. Forsythe LP, Szydiowski V, Murad MH, et al. A systematic review of approaches for engaging patients for research on rare diseases. *Journal of General Internal Medicine* 2014;29(53):788-800.
14. Grut L, Kvam MH. Facing ignorance: people with rare disorders and their experiences with public health and welfare services. *Scandinavian Journal of Disability Research* 2013;15(1):20-32.

For peer review only

For peer review only - http://bmjopen.bmj.com/site/about/guidelines.xhtml

15. Huyard C. What if anything is specific about having a rare disorder? Patients' judgements on being ill and being rare. *Health Expectations* 2009;12(4)
16. Delive L, Samuelsson L, Tallborn A, et al. Stress and well-being among parents of children with rare diseases: a prospective intervention study. *Journal of Advanced Nursing* 2006;53(4):392-402.
17. Farmer JE, Marien WE, Clark MJ, et al. Primary care supports for children with chronic health conditions: identifying and predicting unmet family needs. *Journal of Pediatric Psychology* 2004;29(5):355-67.
18. Crowe A, McKnight A, McAneney H. Communication needs for individuals with rare diseases within and around the healthcare system of Northern Ireland. *Frontiers in Public Health* 2019;7(236)
19. Budych K, Helms TM, C. S. How do patients with rare diseases experience the medical encounter? Exploring the role behavior and its impact on patient-physician interaction. *Health Policy* 2012;105(2-3):154-64.
20. Dudding-Byth T. A powerful team: The family physican advocating for patients with a rare diease. *Australian Family Physician* 2015;44(9):634-38.
21. Baumbusch J, Mayer S, Sloan-Yip I. Alone in a crowd? Parents of children with rare diseases' experiences of navigating the healthcare system. *Journal of Genetic Counseling* 2018;28(1):80-90.
22. Madeo AC, K.E. OB, Bernhardt BA, et al. Factors associated with perceived uncertainty among parents of children with undiagnosed medical conditions. *American Journal of Medical Genetics* 2012;158A(8):1887-84.
23. Watt A, Cameron A, Sturm L, et al. Rapid reviews verus full systematic reviews: an inventory of current methods and practice in health technology assessment. . *International Journal of Technology Assessment in Health Care* 2008;24:133-39.
24. Ganann B, Ciliska D, Thomas H. Expediting systematic reviews: methods and implications of rapid reviews. . *Implementation Science* 2010;5(56)
25. Khangura S, Konnyu K, Cushman R, et al. Evidence summaries: the evolution of a rapid review approach. *Systematic reviews* 2012;1(10)
26. Konnya K, Kwok E, Skidmore B, et al. The effectiveness and safety of emergency department short stay units: a rapid review. *Open Medicine* 2012;6(e10-e16)
27. McMullan J, Crowe A, Downes K, et al. Carer reported experiences: Supporting someone with a rare disease. *Health and Social Care in the Community* 2021;0:1-12.
28. Craig TJ, Banerji A, Riedl MA, et al. Caregivers' role in managing hereditary angioedema and perceptions of treatment-related burden. *Allergy and Asthma Proceedings* 2021;42:S11-S16. doi: 10.2500/aap.2021.42.210029
29. Mooney J, Graham K, Watts RA. Impact of caring for someone with a rare rheumatic condition, views from patients and informal carers-the need for cat-like vigilance. *Rheumatology Advances in Practice* 2019;3(1) doi: 10.1093/rap/rkz003
30. Kanters TA, van der Ploeg AT, Brouwer WBF, et al. The impact of informal care for patients with Pompe disease: An application of the CarerQol instrument. *Molecular Genetics and Metabolism* 2013;110(3):281-86. doi: 10.1016/j.ymgme.2013.07.020
31. Aubeeluck AV, Buchanan H, Stupple EJN. 'All the burden on all the carers': Exploring quality of life with family caregivers of Huntington's disease patients. *Quality of Life Research* 2012;21(8):1425-35.
32. Lyon ME, Thompkins JD, Fratantoni K, et al. Family caregivers of children and adolescents with rare diseases: A novel palliative care intervention. *BMJ Supportive and Palliative Care* 2019;25(25)
33. Somanadhan S, Larkin PJ. Parents' experiences of living with, and caring for children, adolescents and young adults with Mucopolysaccharidosis (MPS). . *Orphanet J Rare Dis* 2016;11(1):138.

For peer review only

34. Wu Y, Sun F, Lee P. Family caregivers' lived experiences of caring for epidermolysis bullosa patients: A phenomenological study. *Journal of Clincial Nursing* 2020;29(9-10):1552-60.
35. Bendixen RM, Houtrow A. Parental reflections on the diagnostic process for duchenne muscular dystrophy: A qualitative study. *Journal of Pediatric Health Care* 2017;31(3):285-92.
36. Rice DB, Canedo-Ayala M, Carboni-Jimenez A, et al. Challenges and support service preferences of informal caregivers of people with systemic sclerosis: a cross-sectional survey. *Disability & Rehabiliation* 2020;42(16):2304-10.
37. Lagae L, Irwin J, Gibson E, et al. Caregiver impact and health service use in high and low severity Dravet syndrome: A multinational cohort study. *Seizure* 2019;65:72-77.
38. Pelentsov LJ, Laws TA, Esterman AJ. The supportive care needs of parents caring for a child with a rare disease: A scoping review. *Disability and Health Journal* 2015;8(4):475-91.
39. Rodriguez AA, Martinez O, Amayra I, et al. Diseases Costs and Impact of the Caring Role on Informal Carers of Children with Neuromuscular Disease. *International Journal of Environmental Research and Public Health* 2021;18(6) doi: 10.3390/ijerph18062991
40. Applebaum AJ, Polacek LC, Walsh L, et al. The unique burden of rare cancer caregiving: caregivers of patients with Erdheim-Chester disease. *Leukemia Lymphoma* 2020;61(6):1406-17.
41. Palacios-Cena D, Famoso-Perez P, Salom-Moreno J, et al. "Living an Obstacle Course": A qualitative study examining the experiences of caregivers of children with Rett Syndrome. *Int J Environ Res Public Health* 2018;16(1):25.
42. Mori Y, Downs J, Wong K, et al. Impacts of caring for a child with the CDKL5 disorder on parental wellbeing and family quality of life. *Orphanet J Rare Dis* 2017;12(1):16.
43. Lopez-Bastida J, Pena-Longobardo LM, Aranda-Reneo I, et al. Social/economic costs and health-related quality of life in patients with spinal muscular atrophy (SMA) in Spain. *Orphanet Journal of Rare Diseases* 2017;12 doi: 10.1186/s13023-017-0695-0
44. Flores D, Ribate MP, Montolio M, et al. Quantifying the economic impact of caregiving for Duchenne muscular dystrophy (DMD) in Spain. *European Journal of Health Economics* 2020;21(7):1015-23. doi: 10.1007/s10198-020-01197-6
45. Rice DB, Carboni-Jimenez A, Canedo-Ayala M, et al. Perceived Benefits and Faciliatators and Barriers to Providing Psychosocial Interventions for Informal Caregivers of People with Rare Diseases: A Scoping Review [Review]. *The Patient: Patient-Centered Outcomes Research* 2020;13(5):471-519.
46. Landfeldt E, Lindgren P, Bell CF, et al. Quantifying the burden of caregiving in Duchenne muscular dystrophy. *Journal of Neurology* 2016;263(5):906-15. doi: 10.1007/s00415-016-8080-9
47. Selman LE, Beynon T, Radcliffe E, et al. 'We're all carrying a burden that we're not sharing': a qualitative study of the impact of cutaneous T-cell lymphoma on the family. *British Journal of Dermatology* 2015;172(6):1581-92. doi: 10.1111/bjd.13583
48. Sloper T, Beresford B. Families with disabled children *BMJ* 2006;333(7575):928-29.
49. Hanbury A, Smith A.B., K B. Deriving Vignettes for the Rare Disease AADC Deficiency Using Parent, Caregiver and Clinician Interviews to Evaluate the Impact on Health-Related Quality of Life. *Patient Related Outcome Measures* 2021;12:1-12.
50. Currie G, Szabo J. “It is like a jungle gym, and everything is under construction”: The parent's perspective of caring for a child with a rare disease. . *Child: Care, Health and Development* 2019;45(1):96-103.
51. Currie G, Szabo J. 'It would be much easier if we were just quiet and disappeared': Parents silenced in the experience of caring for children with rare diseases. *Health Expectations* 2019;22(6):1251-59.
52. Mutch K, Methley A, Hamid S, et al. If they are OK, we are OK: The experience of partners living with neuromyelitis optica. *Disability and Rehabilitation* 2017;39(13):1279-86.

53. McMullan J, Crowe A, Bailie C, et al. Improvements needed to support people living and working with a rare disease in Northern Ireland: current rare disease support perceived as inadequate. *Orphanet J Rare Dis* 2020;15(315)

For peer review only

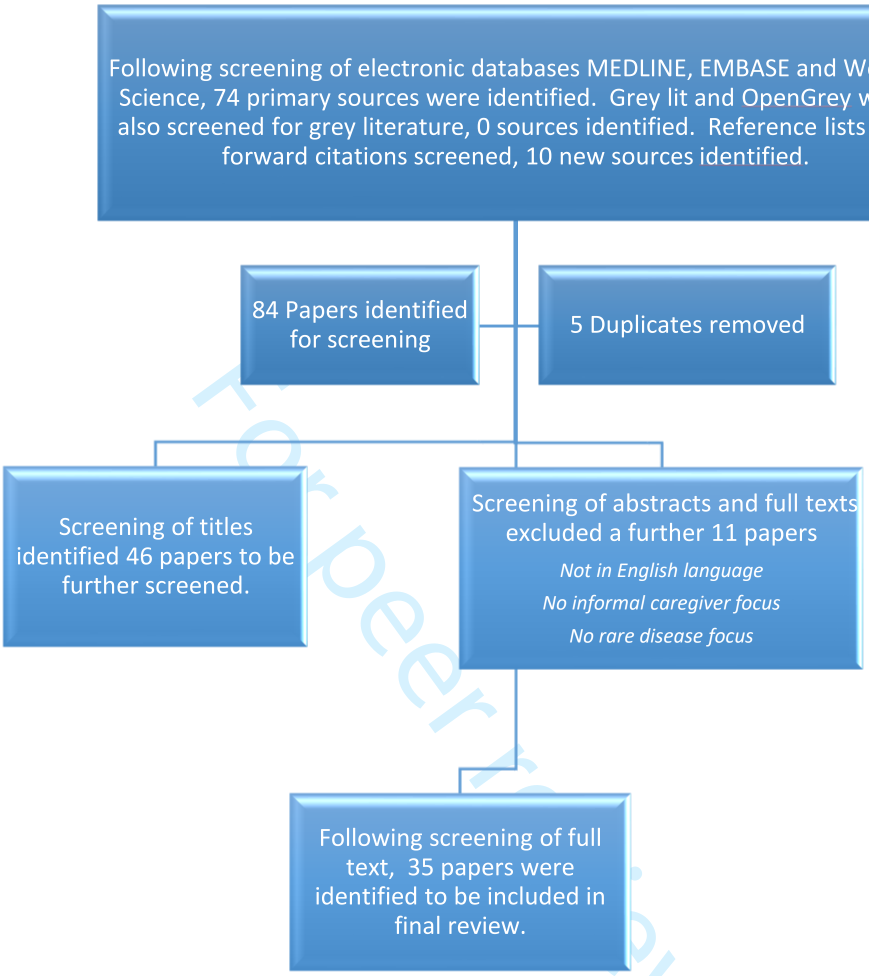

Figure 1 PRISMA flowchart

For peer review only

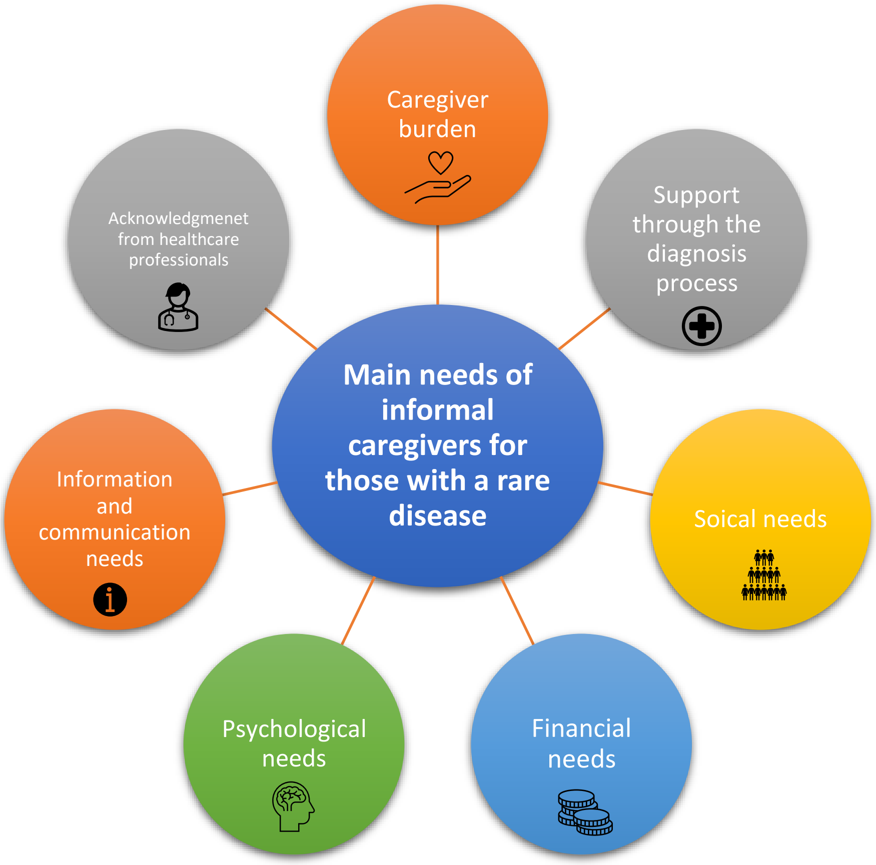

eview only

Figure 2 Main needs of caregivers for those with a rare disease

, Supplementary file 1: Detailed search strategy

‘informal caregiver*’ AND ‘rare disease*’

Non- English articles and duplicates were excluded

For peer review only

Supplementary file 2: Data extraction table

Author & year of publication	Country in which the study was conducted	Data collection method(s)	Participants	Identified caregiver needs
Anderson, M., Elliott EJ, Zurynski YA.<br><br>2009	Australia	Survey	Parents/carers	• Support around diagnosis.<br>• Access to peer support groups.<br>• Psychological support.
Applebaum A.J., Polacek L.C., Walsh L. *et al.*<br><br>2020	USA	Survey and semi-structured interview	Caregivers of patients with Erdheim-Chester disease	• Social connections to prevent extreme isolation
Aubeeluck A.V. Buchanan H.E., Stupple E.J.N.<br><br>2011	England	Focus groups	Caregivers of those with Huntington's Disease	• More time to focus on themselves.
Craig T.J., Banjerji A., Riedl M.A., *et al.*<br><br>2021	USA	Online survey	Adults caring for an individual with Hereditary angioedema (HAE)	• Recognition for the important role they have.<br>• Inclusion in treatment discussions.
Hanbury A, Smith AB & Buesch K<br><br>2021	England	Vignettes were developed	Caregivers	• Engagement with research
Baumbusch, J., Mayer, S., & Sloan-Yip, I.<br><br>2018	Canada	Semi-structured interviews	Parents of children with a RD	• Acknowledgment from healthcare providers<br>• Support coordinating care<br>• Peer support – information and emotional<br>• Policies and programs to validate their role<br>• Support from genetic counsellor to connect them to others

Bendixen, R. M., & Houtrow, A.<br><br>2017	USA	Interviews	Parents of children with Duchenne muscular dystrophy	• Listened to by healthcare providers<br>• Inclusion in the diagnostic process<br>• Improved delivery of the diagnosis<br>• Improved follow up after diagnosis
Cañedo-Ayala, M., Rice, D. B., Levis, B., et al.<br><br>2020	USA	Questionnaire	Informal caregivers	• Mental health support
Currie, G., & Szabo, J.<br><br>2019	Canada	Semi-structured interviews	Parents of children with RDs	• Communication issues need addressed as parents often know more about the disease than healthcare providers.<br>• Improved coordination of care between providers and services caring for children with rare diseases.<br>• Gap in accessibility to government supports needs addressed.
Currie, G., & Szabo, J.<br><br>2019	Canada	Semi-structured interviews	Parents of children with rare neurodevelopmental diseases	• Health-care providers need more understanding of rare conditions.<br>• More support needed, parents often 'lose themselves' in this demanding caring role.
Flores D., Ribate M.P., Montolio M., *et al.*<br><br>2020	Spain	Questionnaires	Caregivers to patients with Duchenne muscular dystrophy	• Financial support.
Kanters T.A., van der Ploeg Ans. T, Brouwer W.B.F., *et al.*<br><br>2013	Netherlands	Questionnaire	Caregivers for those with Pompe disease.	• Support needed for informal caregivers.
Kasparian, N. A., Rutstein, A., Sansom-Daly, *et al.*	Australia	Telephone interviews	Patients and caregivers affected by Von Hippel-Lindau disease	• More supportive care services requested.

For peer review only

2015				
Landfelt E., Lindgren P., Bell C.F., *et al.*<br><br>2016	Germany, Italy, UK, USA	Visual analogue scale, survey, interview	Caregivers to patients with Duchenne muscular dystrophy	• Screening for depression.<br>• Holistic approach to family mental health needed.
Lagae, L., Irwin, J., Gibson, E., *et al.*<br><br>2019	Europe	Survey	Caregivers for those with Dravet syndrome (DS)	• Time needed to escape caring duties.<br>• Formal support and respite required.<br>• Stress is common – support needed.
Lopez-Bastida J., Pena-Longobardo L.M., Aranda-Reneo I, *et al.*<br><br>2017	Spain	Questionnaire	Patients and caregivers with Spinal Muscular Atrophy	• Financial support<br>• QoL must be addressed
Lyon, M. E., Thompkins, J. D., Fratantoni, K., *et al.*<br><br>2019	USA	Semi-structured interviews	Caregiving families	• Worries about the future need to be addressed.<br>• More time for themselves needed.<br>• Financial concerns must be addressed.
McKnight, A. J. M., Walker, R., Collins, C. (2020).	Northern Ireland	Report	N/A	• Access to accurate information<br>• Access to appropriate services<br>• Improved communication
McMullan, J., Crowe, A. L., Bailie, C. *et al.*<br><br>2020	Northern Ireland	Survey and semi-structured interviews	Rare disease collaborative groups	• Caregivers often overlooked in RD research – their opinions and experiences must be valued.
McMullan J, Crowe A.L., Downes K., *et al.*<br><br>2021	Northern Ireland	Survey and workshop	Caregivers of those with a rare disease	• Improved interactions with healthcare professionals.<br>• Improved emotional, psychological and social support.<br>• Assistance with finances.<br>• Better awareness of support services.
Mooney J., Graham K. & Watts R.A.	England	Semi-structured interviews	Patients with ANCA-associated vasculitis and their caregivers.	• Emotional support is needed.<br>• Reassurance about the future.

For peer review only

2019				
Mori, Y., Downs, J., Wong, K., *et al.*<br><br>2017	Australia	Survey	Families with a child with CDKL5 disorder	• Burden of daily caregiving requires support, particularly in relation to emotional wellbeing, sleep problems, financial difficulties, QoL.
Mutch, K., Methley, A., Hamid, S., *et al.*<br><br>2017	England	Semi-structured interviews	Partners of people with NMO	• Acknowledgement from HCP regarding the vital role they play in caring.
Palacios-Cena, D., Famoso-Perez, P., Salom-Moreno, J., *et al.*<br><br>2018	Spain	Interviews, focus groups, researcher's field notes, caregiver's personal documents	Caregivers of children with Rett Syndrome	• Answers needed regarding 'the first symptoms' and 'the need for a diagnosis'.<br>• Help with managing day to day life.<br>• Financial support.
Pelentsov, L. J., Fielder, A. L., & Esterman, A. J.<br><br>2016	Australia	Semi-structured focus group interviews	Parents of a child with a RD	• Social isolation must be addressed.<br>• Knowledge of HCP needs to be improved.<br>• Support needed as family relationship often impacted due to demands of caring.
Pelentsov, L. J., Laws, T. A., & Esterman, A. J.<br><br>2015	Australia	Scoping Review	Parents of child with a RD	• Improved parental supportive care – common unmet needs between RDs.
Rice D.B., Canedo-Ayala M., Carboni-Jimeniez A., Carrier M-E, *et al.*<br><br>2020	USA	Online questionnaire	Caregivers of people with SSc	• Emotional support is required.<br>• Help with physical needs.<br>• Interventions needed delivered through hardcopy or online resources, including those delivered after the care recipient's diagnosis.
Rice D.B., Carbino-Jimeniz A., Canedo-Ayala M. *et al.*<br><br>2020	USA	Scoping review	N/A	• Psychosocial interventions needed to reduce caregivers stress, burden and feelings of isolation among caregivers.

For peer review only

				• Future research should design interventions for caregivers.
Rodriguez A.A., Martinez O., Amayra I, *et al.*<br><br>2021	Spain	Questionnaire	Carers of children with neuromuscular disease	• Financial assistance.<br>• Employment support.<br>• Physical and psychological support.
Selman L.E., Beynon T., Radcliffe S., *et al.*<br><br>2014	London	Semi-structured qualitative interviews	Adult informal caregivers of patients with Cutaneous T-cell lymphoma	• Easily accessible services are needed that include the family in the unit of care, provide support and information, and understand the process of family adjustment and adaptation.
Sloper, T., & Beresford, B.<br><br>2006	UK	Report	Families of disabled children	• Policies and structures to support social and economic needs.
Somanadhan, S., & Larkin, P. J.<br><br>2016	Ireland	In-depth interviews	Parents of those with Mucopolysaccharidosis	• Reassurance and certainty needed about the future.
Wiblin, L., Durcan, R., Lee, M., *et al.*<br><br>2017	England	Qualitative in depth interviews	Patients and caregivers living with MSA and PSP	• Better connections to others to reduce social isolation.<br>• Improved communication.
Williams, J. K., Skirton, H., Paulsen, J. S., *et al.*<br><br>2009	USA, Canada	Focus groups	Adult caregivers of people with Huntington's disease	• Emotional distress support should be provided.<br>• Assistance in managing several roles.<br>• Mental health monitoring.
Wu, Y., Al-Janabi, H., Mallett, A., *et al.*<br><br>2020	Australia	Clinical data was used from Mitochondrial Disease, Epileptic	Parents of those with rare genetic conditions	• Health effects on family members need to be considered.

For peer review only

		Encephalopathy and Brain Malformation projects		

For peer review only
